# Loss of Neogenin alters branchial arch development and leads to craniofacial skeletal defects

**DOI:** 10.3389/fcell.2024.1256465

**Published:** 2024-02-09

**Authors:** Sabrina Quilez, Emilie Dumontier, Christopher Baim, Joseph Kam, Jean-François Cloutier

**Affiliations:** ^1^ The Neuro—Montreal Neurological Institute and Hospital, 3801 University, Montréal, QC, Canada; ^2^ Department of Neurology and Neurosurgery, McGill University, Montréal, QC, Canada; ^3^ Department of Anatomy and Cell Biology, McGill University, Montréal, QC, Canada

**Keywords:** neogenin, mandible, apoptosis, osteoblast differentiation, gene expression, BMP, branchial arch, craniofacial

## Abstract

The formation of complex structures, such as the craniofacial skeleton, requires precise and intricate two-way signalling between populations of cells of different embryonic origins. For example, the lower jaw, or mandible, arises from cranial neural crest cells (CNCCs) in the mandibular portion of the first branchial arch (mdBA1) of the embryo, and its development is regulated by signals from the ectoderm and cranial mesoderm (CM) within this structure. The molecular mechanisms underlying CM cell influence on CNCC development in the mdBA1 remain poorly defined. Herein we identified the receptor Neogenin as a key regulator of craniofacial development. We found that ablation of Neogenin expression via gene-targeting resulted in several craniofacial skeletal defects, including reduced size of the CNCC-derived mandible. Loss of Neogenin did not affect the formation of the mdBA1 CM core but resulted in altered *Bmp4* and *Fgf8* expression, increased apoptosis, and reduced osteoblast differentiation in the mdBA1 mesenchyme. Reduced BMP signalling in the mdBA1 of Neogenin mutant embryos was associated with alterations in the gene regulatory network, including decreased expression of transcription factors of the Hand, Msx, and Alx families, which play key roles in the patterning and outgrowth of the mdBA1. Tissue-specific Neogenin loss-of-function studies revealed that Neogenin expression in mesodermal cells contributes to mandible formation. Thus, our results identify Neogenin as a novel regulator of craniofacial skeletal formation and demonstrates it impinges on CNCC development via a non-cell autonomous mechanism.

## Introduction

Tissue growth and morphogenesis relies on signalling between various populations of cells in developing structures that regulate cell survival, proliferation, and differentiation (Reh, 1987; Eisen, 1992; Artavanis-Tsakonas et al., 1999; Zhang et al., 2013). During craniofacial development, intimate interactions between the cranial mesoderm (CM), and cranial neural crest cells (CNCCs) contribute to the formation of bones of the head. While CM-derived cells give rise to bones of the posterior region of the head, such as the interparietal and parietal bones, CNCCs give rise to anterior bones, including the nasal bone and mandible. In certain regions, such as in the mandibular part of the first branchial arch (mdBA1) that gives rise to the mandible, CM cells and CNCCs have close spatial interactions and reciprocal signaling activities that play important roles for the development of CNCC-derived chondrocytes and osteoblasts that form the mandible (Noden and Trainor, 2005; Fan et al., 2016).

CNCCs delaminate from the dorsal region of the neural tube and migrate to populate the frontonasal process and the branchial arches, where they proliferate and form mesenchymal progenitors of bone and cartilage. The development of CNCCs is affected by a complex network of transcription factors in the developing mdBA1, whose expression is regulated by several morphogens that act in an autocrine or paracrine fashion, including Bone Morphogenetic Proteins (BMPs), Fibroblast growth factors (FGFs), endothelin1 (Edn1), and Sonic Hedgehog (SHH) (Solloway and Robertson, 1999; Trumpp et al., 1999; Jehong et al., 2004; Ozeki et al., 2004; Liu et al., 2005; Tavares et al., 2012; Xu et al., 2019).

The CM is proposed to play an important role in the development of CNCCs. Altered architecture of the CM in mesoderm-specific *Twist1* mutant mice is associated with severe craniofacial bone defects (Bildsoe et al., 2013). Ablation of the transcription factor TBX1 in the mesoderm leads to changes in the pattern of expression of key morphogens in ectodermal cells of the mdBA1, including BMP4 and FGF8, resulting in reduced size of the CNCC-derived mandible (Zhang et al., 2006; Aggarwal et al., 2010). Hence, the mesoderm can modulate CNCC development by influencing expression of ectoderm-derived signals but could also provide signals that directly affect CNCC development (Fan et al., 2016).

The dosage of BMP signalling in the mdBA1 is particularly important for the regulation of transcriptional programs necessary for mandible formation. Indeed, both reduced and enhanced BMP signalling in the mdBA1 lead to alterations in the gene regulatory network of the mdBA1 and to reduced mandible size (Solloway and Robertson, 1999; Liu et al., 2005; Ko et al., 2007; Bonilla-Claudio et al., 2012). In other systems, the level of BMP signalling has been proposed to be modulated by Neogenin (Neo1), a cell surface receptor of the immunoglobulin superfamily of proteins, which can bind to multiple families of ligands, including Netrins, Repulsive Guidance Molecules (RGMs), and some BMPs (Keeling et al., 1997; Wang et al., 1999; Monnier et al., 2002; Matsunaga et al., 2004; Rajagopalan et al., 2004; Hagihara et al., 2011). Whether Neogenin can modulate BMP signalling during mdBA1 and craniofacial development remains unknown. In long bone outgrowth, an association between Neogenin and RGMb, a BMP co-receptor, can promote BMPR recruitment to lipid-rich membrane microdomains, increasing BMP2 signalling and chondrocyte differentiation (Samad et al., 2005; Zhou et al., 2010). In contrast, direct binding of BMP2 to Neogenin inhibits BMP-dependent osteoblastic differentiation of C2C12 cells, suggesting that Neogenin can negatively regulate BMP signalling (Hagihara et al., 2011). Thus, Neogenin appears to have differential effects on BMP signalling during skeletal formation in a context-dependent manner and may therefore play a role in craniofacial development.

Herein, we used mouse loss-of-function studies to examine the contribution of Neogenin to craniofacial skeletal development. We find that loss of Neogenin expression leads to defects in several craniofacial bones, including the mdBA1-derived mandible. Altered expression of *Fgf8* and *Bmp4* in the mdBA1 ectoderm of *Neo1* ^*−/−*^ embryos is associated with reduced BMP signalling within this structure, and with reduced mandible size. Interestingly, Neogenin expression in mesodermal cells contributes to mdBA1 morphogenesis and mandible formation, suggesting it may impinge upon CNCC development by modulating expression of mesoderm-derived signals. Taken together, these findings identify a new function for Neogenin as a crucial regulator of craniofacial development.

## Materials and methods

### Animals

The *Neogenin* (*Neo1*) null and conditional (*Neo1-lox*) (Kam et al., 2016), *Mesp1-Cre* (Saga et al., 1999), *Wnt1-Cre* (Danielian et al., 1998; Rowitch et al., 1999), and *Gt(ROSA)26Sor* ^*tm1Sor*^(*R26R*) (Soriano, 1999) mouse lines have been described and were backcrossed into the C57Bl6J background for at least three generations. For timed pregnancies, morning of the vaginal plug was considered E0.5. All animal procedures have been approved by the Montreal Neurological Institute Animal Care Committee and McGill University, in accordance with the guidelines of the Canadian Council for Animal Care.

### Skeletal preparations and staining

Whole-embryo skeletal preparations and staining with Alcian blue and Alizarin red to label cartilage and bone, respectively, were previously described (Marcil et al., 2003).

### Immunohistochemistry

Embryos were dissected, immersion fixed in 4% paraformaldehyde (PFA), and processed for immunohistochemistry using conditions previously described (Cho et al., 2012). For all antibodies, sections were incubated overnight with primary antibody at 4°C using the following dilutions: goat anti-Neogenin, 1:250 (R&D Systems), mouse anti-β-galactosidase (β-gal), 1:500 (Promega), rat anti-CD31, 1:200 (BD Pharmingen), rabbit anti-cleaved caspase-3 (CC3), 1:1000 (Cell Signaling Technology), rabbit anti-Phosphohistone H3 (PHH3), 1:1000 (EMD Millipore), mouse anti-Islet1, 1:80 (DSHB), mouse anti-Runx2, 1:5 (DSHB), rabbit anti-Col2a1, 1:350 (Origene). After rinsing in PBS, primary antibody binding was detected with the appropriate fluorescent conjugated secondary antibody (1:500) (Invitrogen). For Islet-1 and Runx2 staining, an antigen retrieval step was added before blocking where slides were incubated with 0.01M sodium citrate (pH 6.0) for five to ten minutes on a hot plate maintained at 95°C.

### Phospho-SMAD1/5/8 immunohistochemistry

E10.5 embryos were dissected and fixed in 4% PFA on a rocker at 4°C for 1.5 h. Embryos were then placed in a 10 mM sodium citrate buffer (pH 6.0) overnight at 4°C. Whole embryos were transferred to fresh sodium citrate buffer and heated to 95°C for 5 min to promote antigen retrieval and then cryopreserved in 30% sucrose and embedded in OCT for freezing. Embryonic sections were processed as described above and incubated with a Phospho-SMAD1/5/8 antibody, 1:1000 (New England Biolabs) followed by detection with a biotinylated secondary antibody and Alexa488-conjugated streptavidin (Invitrogen).

### Image capture and analysis

Fluorescence and brightfield images of processed sections were obtained using the Zeiss Axio Imager M1 with Eclipse (Empix Imaging) and Zeiss ZenPro imaging software, and analyzed using ImageJ and Icy. The average number of CC3 or PHH3-positive cells per mm^2^ was calculated by measuring CC3 or PHH3-positive cell density in each section of the left and right mdBA1 at E9.5-11.5. For quantifications of CC3 and PHH3 density at E12.5, only CC3 and PHH3-positive cells within Col2a1-or Runx2-positive regions were counted. Statistical significance was assessed using students’ t-tests or two-way ANOVA analyses performed using Prism software (GraphPad). Skeletal preparations were imaged on a ZEISS SteREO Discovery.V20 with Zeiss AxioVision 4.8 imaging software. The lengths and thicknesses of Meckel’s cartilage (MC) and of the mandibular bone were quantified using ImageJ. Lengths of dissected mandibles were measured as the longest distance between the proximal and distal extremities for both structures. Due to the irregular shapes of both the MC and of the mandibular bone, to obtain a measurement of the average thickness, we measured the visible surface area of these structures when laid flat, divided by their length.

### Whole-mount X-gal

E10.5 embryos were fixed for 12 min in 4% PFA on ice, and then rinsed twice in PBS. X-gal staining was performed as previously described (Cho et al., 2011). Embryos were dehydrated and cleared using a series of glycerol dilutions (20%, 40%, 60%, and 80%) in PBS.

### Wholemount *in situ* hybridization

Whole mount *in situ* hybridization staining on E9.5 and E10.5 embryos was performed as previously described (Marcil et al., 2003). cDNAs for preparation of the cRNA probes were kindly provided by Dr. Loydie Jerome-Majewska (*Tbx1*), Dr. Anthony Firulli (*Smad6*, *Hand2*, *Gata3*, and *Dlx5*), Dr. Takako Makita (*Edn1*), Dr. Licia Selleri (*Alx1*, *Alx4*), Dr. Jacques Drouin (*Dlx3*, *Msx2*, *Fgf8, Pitx2*), Dr. Samantha Butler (*Bmp4*), and Dr. Eric Olson (*Hand1*)*.*

### qRT-PCR

Total RNA was extracted from dissected mdBA1 of E10.5 or E11.5 embryos using the Qiagen RNeasy Kit and cDNA libraries were generated using Superscript (Invitrogen). Quantitative PCR was performed using Fast Sybr Green Master Mix (Invitrogen) on a StepOne Plus thermocycler (Applied Biosystems). Relative changes in mRNA levels normalized to *Hprt1* expression were quantified for control and *Neo1* ^*−/−*^ samples.

## Results

### Neogenin ablation leads to craniofacial malformations

**FIGURE 1:**
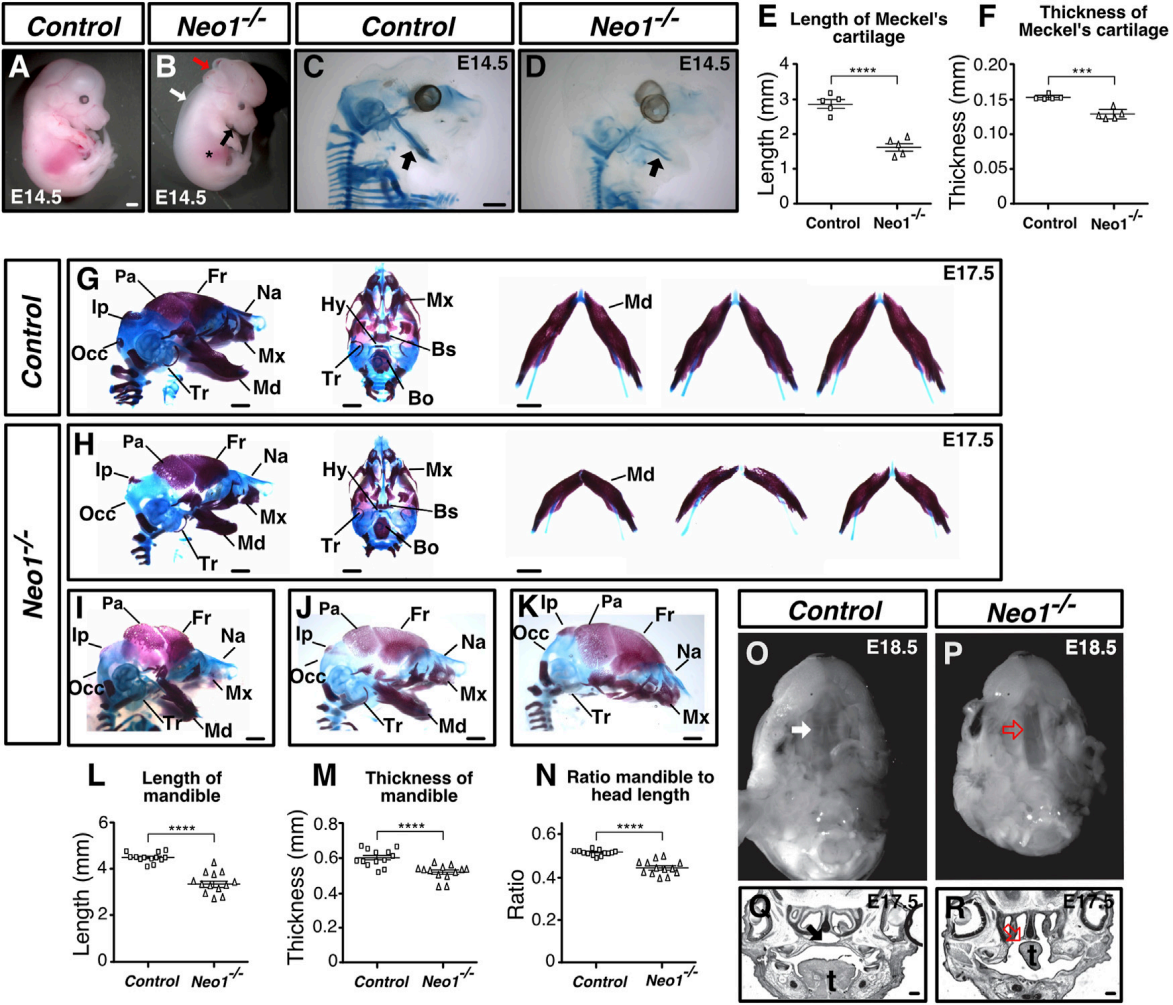
Neogenin ablation leads to craniofacial bone defects. **(A, B)** E14.5 *Neo1* ^*−/−*^ embryos display a shorter jaw (black arrow), hypomorphic liver (asterisk), edema (white arrow), and exencephaly defects (red arrow). **(C, D)** Alcian blue (cartilage) staining of E14.5 embryos to highlight Meckel’s cartilage (arrow). **(E, F)** Quantification of the length **(E)** and thickness **(F)** of Meckel’s cartilage revealed a shorter and thinner structure in *Neo1* ^*−/−*^ embryos. (n = 5 control and *Neo1* ^*−/−*^ embryos; Student’s unpaired *t*-test, ****p < 0.0001, ***p < 0.001, Bars on graphs indicate mean ± s. e.m.). **(G, H)** Alizarin red (bone) and Alcian blue (cartilage) staining of E17.5 control **(G)** and *Neo1* ^*−/−*^ **(H)** embryos from lateral (left) and ventral (middle) views. The lateral view illustrates the micrognathia while the horizontal view of the dissected mandible from three representative embryos (right) shows reduced mandible thickness (quantified in **L**, **N** and **M**, respectively) in *Neo1* ^*−/−*^ embryos (n = 13 control and *Neo1* ^*−/−*^ embryos, Student’s unpaired *t*-test, ****p < 0.0001; Bars on graphs indicate mean ± s. e.m). Improper formation of additional CNCC-derived bones, including the nasal, maxillary, and tympanic bones is observed in *Neo1* ^*−/−*^ embryos. Several CM-derived skull bones are also affected, including the parietal, interparietal, and occipital bones. **(I–K)** Phenotypic variability of craniofacial defects observed in *Neo1* ^*−/−*^ embryos, with some embryos showing a more severe reduction in parietal bone size **(I, J)** or the absence of a mandible **(K)**. **(O, P)** Ventral view of E18.5 heads after removal of the mandible reveals a cleft palate in *Neo1* ^*−/−*^ animals (red open arrow). **(Q, R)** Nissl staining of coronal sections of E17.5 heads show improper closure of the palatal shelf (red open arrow) and tongue (t) agenesis in *Neo1* ^*−/−*^ embryos. Bones, or their expected location when missing, are labelled as follows: Bo: basioccipital, Bs: basisphenoid, Fr: frontal, Hy: hyoid, Ip: interparietal, Md: mandible, Mx: maxillary, Na: nasal bone, Occ: occipital, Pa: parietal, Tr: tympanic ring. Scale bars: 1 mm **(A–D and G–K)**, 200 μm **(Q, R)**.

We have previously reported a loss-of-function *Neo1* allele bearing defects in neurogenesis within the developing olfactory epithelium (Kam et al., 2016). Our examination of *Neo1* ^*−/−*^ embryos revealed several morphological defects that we have yet to report, including a shorter mandible, a hypomorphic liver, edema, and exencephaly with variable penetrance (Figures 1A,B; Supplementary Table S1). An analysis of the size of *Neo1* ^*−/−*^ embryos at various time points of embryonic development indicated that the overall growth of these embryos between E9.5 and E13.5 appeared similar to control embryos, but that *Neo1* ^*−/−*^ embryos were smaller than control embryos by E14.5 (Supplementary Table S2). Since a noticeably shorter mandible was observed in *Neo1* ^*−/−*^ embryos by E14.5 (Figure 1B), we examined more closely the craniofacial bones in these embryos by performing bone and cartilage staining using Alizarin red and Alcian blue staining, respectively, in E14.5 and E17.5 embryos. At E14.5, we observed overall less cartilage in *Neo1* ^*−/−*^ embryos, including in the head, as well as a shorter and thinner Meckel’s cartilage (Figures 1D–F). Our analysis at E17.5 showed defects in several bones of the head, which varied across *Neo1* ^*−/−*^ embryos examined (Figures 1H–K). While the majority of embryos showed reduced bone size (Figures 1H,I), some embryos displayed a complete absence of the mandible (Figure 1K). The defects were observed in bones that originate from either the CM or CNCCs. The CM-derived interparietal and parietal bones appeared underdeveloped in some *Neo1* ^*−/−*^ embryos (Figures 1H–J). Several CNCC-derived bones were also underdeveloped, including the mandible, tympanic ring, maxillary, and nasal bones (Figures 1H–K). The reduced mandible length was observed in 77% (54/70) of *Neo1* ^*−/−*^ embryos analyzed by Alizarin red and Alcian blue staining or by visual examination of dissected embryos showing this phenotype (Figures 1H–J, Supplementary Table S1). Furthermore, an analysis of the ratio of mandible length to the overall length of the skull confirmed that the reduced size of the mandible is not solely accounted for by the reduced size of the *Neo1* ^*−/−*^ embryos at E17.5 (Figure 1N). In contrast to the shorter mandible observed in *Neo1* ^*−/−*^ embryos, the development of another neural crest-derived structure, the dorsal root ganglia, is normal in these embryos, indicating that loss of Neogenin does not have a widespread deleterious effect on all neural crest-derived structures (Supplementary Figure S1). An examination of the palate revealed that the palatal bone is unfused in *Neo1* ^*−/−*^ embryos leading to a cleft palate in 80% of the embryos analyzed (4/5) (Figures 1P,R; Supplementary Table S1). Hence, ablation of Neogenin expression results in multiple defects in craniofacial bones, including a shortened mandible.

### Neogenin is expressed in the mdBA1 during development

**FIGURE 2:**
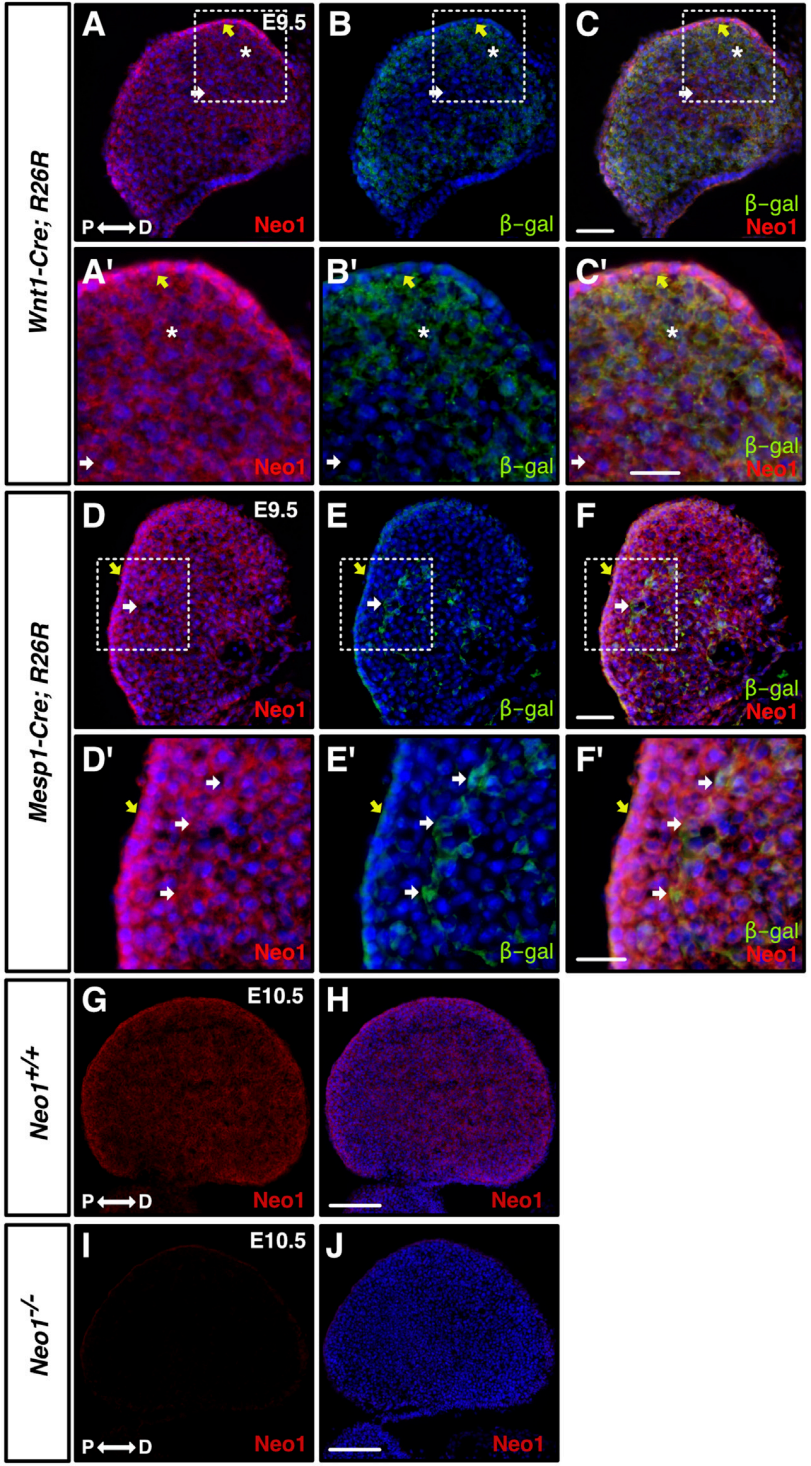
Neogenin is expressed in CNCCs and in the mdBA1 mesodermal core. **(A–F′)** Transverse sections from E9.5 embryos expressing β-galactosidase in neural crest-derived [*Wnt1-Cre; R26R*, **(A–C′)**] or mesoderm-derived [*Mesp1-Cre; R26R*, **(D–F′)**] cells, immunolabelled with antibodies against Neogenin (red) and β-galactosidase (green) and counterstained with Hoechst 33,342 (blue). Zoom-in views of the mdBA1 show high levels of Neogenin expression in β-galactosidase-positive CNCCs (asterisk) **(A′–C′)** and mesodermal cells **(D′–F′)** (white arrow). Neogenin is also expressed in the ectoderm (yellow arrow). **(G–J)** Neogenin expression in the mdBA1 is maintained at E10.5 **(G, H)**. Neogenin signal was not observed in sections from E10.5 *Neo1* ^−/−^ embryos, demonstrating the specificity of the Neogenin antibody **(I, J)**. P: proximal; D: distal. Scale bars: 50 μm **(A–F)**; 100 μm **(A′–F′)**; **(G–J)**.

Since a reduced mandible size is the most consistent phenotype observed in the craniofacial bones of *Neo1* ^*−/−*^ embryos, we next focused our analyses on whether the development of the mdBA1, which gives rise to the mandible, is affected in these embryos. We first investigated the expression of Neogenin in the developing mdBA1 by immunohistochemistry using a Neogenin-specific antibody that we have characterized (Figures 2G–J) (Kam et al., 2016). We examined its expression in mdBA1 from E9.5 embryos expressing the β-galactosidase protein in either CNCCs (*Wnt1-Cre; R26R*) or CM-derived cells (*Mesp1-Cre; R26R*). At E9.5, Neogenin is expressed in CNCCs, CM-derived cells, as well as in the ectoderm (Figures 2A–F’). The expression of Neogenin is maintained in these three populations of cells at E10.5 and E11.5 (Figure 2G, data not shown). Hence, these experiments demonstrated that Neogenin is highly expressed in three populations of cells that contribute to the development of the mdBA1.

### Loss of Neogenin leads to a transient increase in apoptosis in the mdBA1 mesenchyme and to reduced osteoblastic differentiation

**FIGURE 3:**
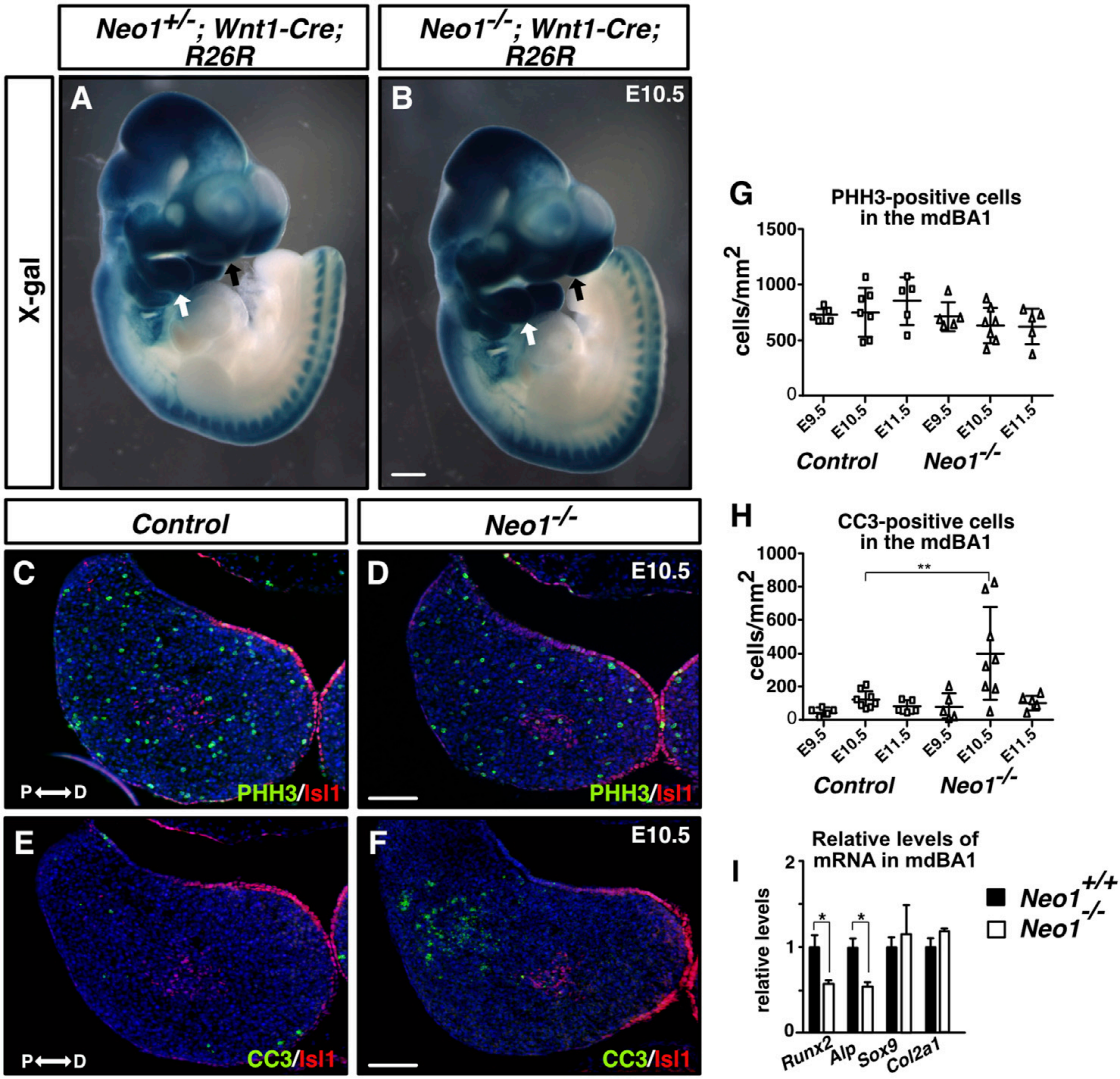
Ablation of Neogenin leads to a transient increase in levels of apoptosis in the proximal region of the mdBA1. **(A, B)** Whole-mount X-gal staining of E10.5 *Neo* ^*+/−*^ *; Wnt1-Cre; R26R* **(A)** or *Neo1* ^*−/−*^ *; Wnt1-Cre; R26R* **(B)** embryos. β-galactosidase-positive CNCCs invade the mdBA1 (white arrow) and frontonasal process (black arrow) in control and mutant embryos (n = 6 *Neo* ^*+/−*^ *; Wnt1-Cre; R26R* and 3 *Neo1* ^*−/−*^ *; Wnt1-Cre; R26R*). **(C–F)** Transverse mdBA1 sections from control and *Neo1* ^−/−^ E10.5 embryos immunolabelled with antibodies marking distal ectoderm and core mesoderm cells (Islet1 (Isl1), red) and co-immunolabelled with antibody markers of either proliferation (phoshospho-histone H3 (PHH3), green) **(C, D)** or cell death (cleaved caspase-3 (CC3_, green) **(E, F)**, and counterstained with Hoechst 33,342 (blue). **(G, H)** Quantification of the density of PHH3- **(G)** and CC3-positive cells **(H)** in mdBA1 sections of E9.5, E10.5, and E11.5 embryos. A significant increase in the number of cells undergoing apoptosis was found in the proximal region of the mdBA1 in *Neo1^−/−^* embryos at E10.5 **(F, H)** (controls: n = 5 at E9.5, 8 at E10.5 and 5 at E11.5; *Neo1^−/−^*: n = 5 at E9.5, 8 at E10.5, and 5 at E11.5; Two-way ANOVA test followed by a Bonferroni’s multiple comparisons test, **p < 0.01 at E10.5, Bars on graphs indicate mean ± s. e.m.). P: proximal; D: distal. Scale bars: 500 μm **(A, B)**, 100 μm **(C–F)**. **(I)** qRT-PCR quantification of relative levels of mRNA markers of osteogenic (*Runx2* and *Alp*) and chondrogenic (*Sox9* and *Col2a1*) differentiation showed a reduction in the expression of *Runx2* and *Alp* in the mdBA1 of *Neo1^−/−^* embryos. (n = 4 control and 4 *Neo1^−/−^*, Student’s unpaired *t*-test, *p < 0.05, Bars on graphs indicate mean ± s. e.m.).

**FIGURE 4:**
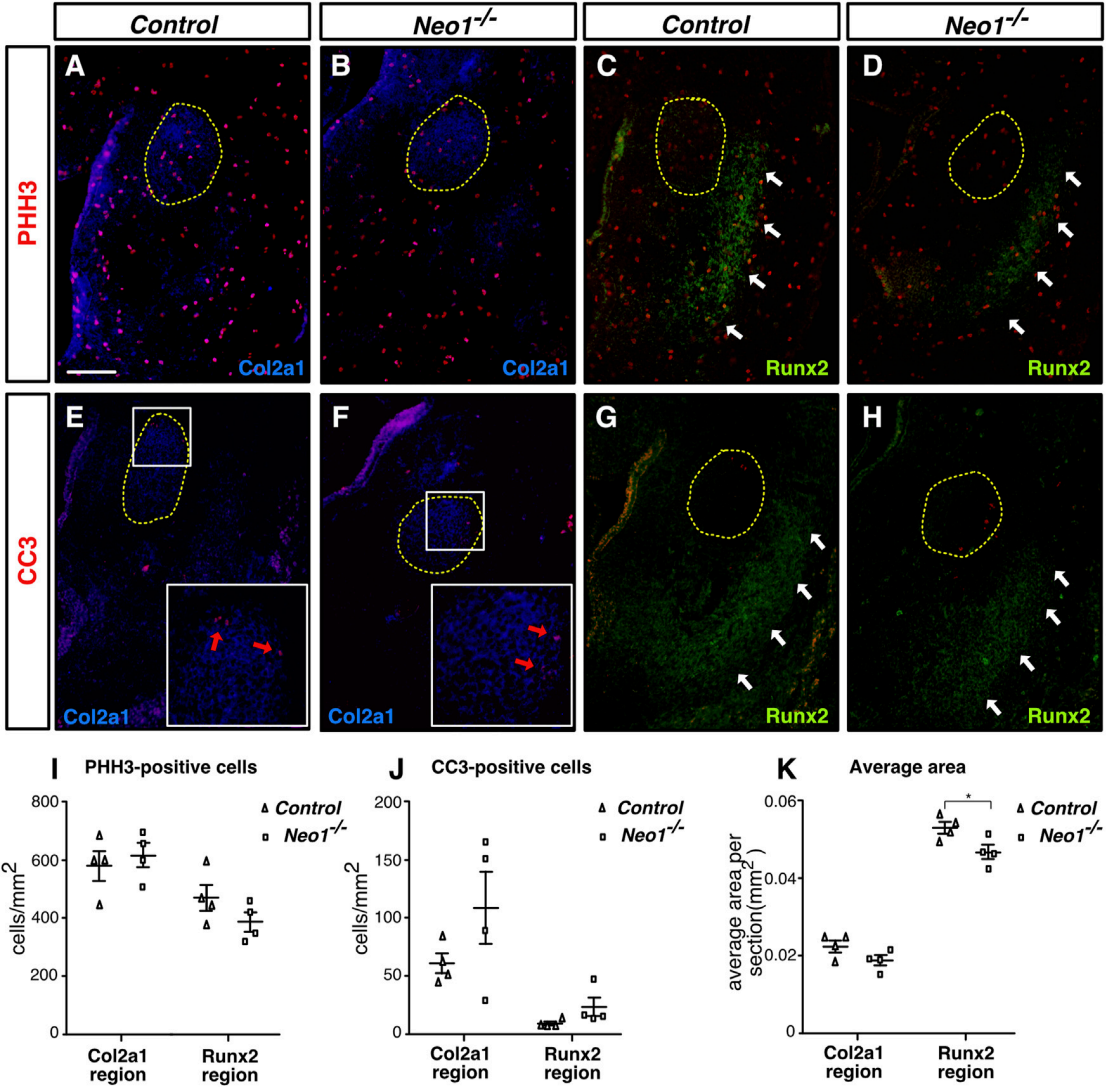
Ablation of Neogenin does not significantly impact cell death or cell proliferation in regions of early bone and cartilage differentiation. **(A–H)** Coronal mdBA1 sections from control and *Neo1^−/−^* E12.5 embryos immunolabelled for markers of chondrocytes (Col2a1 in blue **(A,B,E, F)** and osteoblasts (Runx2 in green **(C,D,G, H)**, respectively, and co-immunolabelled for markers of either cell proliferation (phoshospho-histone H3 (PHH3), red) **(A–D)** or cell death (cleaved caspase-3 (CC3), red) **(E–H)**. **(I, J)** Quantification of the density of PHH3- **(I)** or CC3- **(J)** positive cells within Col2a1-or Runx2-positive regions. **(K)** Quantification of the average size of Col2a1- or Runx2-positive regions per section. A significant decrease in the size of the Runx2-positive area was observed in sections of the mdBA1 in *Neo1^−/−^* embryos. The developing Meckel’s cartilage is circled in yellow **(A–H)**, white arrows point to Runx2-positive areas **(C,D,G, H)**, and red arrows point to CC3 positive cells **(E, F)**. (n = 4 controls and 4 *Neo1* ^*−/−*^; Student’s unpaired *t*-test, *p < 0.05; Bars on graphs indicate mean ± s. e.m.). Scale bars: 100 μm **(A–H)**.

The reduced mandible size observed in *Neo1* ^*−/−*^ embryos could arise from improper development of CNCCs, which rely on extrinsic signals from the mesodermal core and ectoderm to survive, proliferate, and undergo differentiation. CNCCs are specified in the dorsal neural tube from which they delaminate and migrate into the branchial arches. In the mdBA1, they proliferate and undergo differentiation into chondrocytes and osteoblasts to form Meckel’s cartilage and the bone of the mandible, respectively. To examine the migration of CNCCs, we used the *Wnt1-Cre* reporter system (*Wnt1-Cre; R26R*) to visualize neural crest cell migration in whole-mount X-gal staining of control and *Neo1* ^*−/−*^ embryos. The migration of CNCCs to the mdBA1 and frontonasal process did not appear to be grossly affected in *Neo1* ^*−/−*^ *; Wnt1-Cre; R26R* embryos indicating that the reduced mandibular size observed in *Neo1* ^*−/−*^ embryos is unlikely due to severe defects in CNCC migration (Figures 3A,B). We next examined cell proliferation and survival in the mdBA1 mesenchyme by immunostaining with antibodies for the mitosis and apoptosis markers, phospho-histone H3 and cleaved caspase-3, respectively. We did not observe a significant change in the number of phospho-histone H3-positive cells in the mdBA1 of *Neo1* ^*−/−*^ embryos at the three embryonic stages analyzed (Figures 3C,D,G). Interestingly, a transient increase in apoptosis was observed in the proximal part of the mdBA1 in E10.5 *Neo1* ^*−/−*^ embryos, which is not observed at E9.5 and E11.5 (Figures 3E,F,H). Most cells undergoing apoptosis were observed outside the Islet1-positive mesodermal core, indicating they are likely CNCCs. To assess whether the differentiation of osteoblasts and chondrocytes is affected in *Neo1* ^*−/−*^ embryos, we measured the levels of expression of osteoblast (*Runx2*, *Alp*) and chondrocyte (*Sox9*, *Col2a1*) differentiation markers in the mdBA1 at E11.5. While the levels of *Sox9* and *Col2a1* were unchanged in the mdBA1 of *Neo1* ^*−/−*^ embryos, the expression of *Runx2* and *Alp* was decreased, indicating reduced differentiation of CNCCs into osteoblasts (Figure 3I). In keeping with the reduced osteoblastic differentiation observed at E11.5, immunohistochemical analyses of the developing mandible at E12.5 revealed a decrease in the size of the Runx2-positive area around the Meckel’s cartilage in *Neo1* ^*−/−*^ embryos, which is not due to reduced cellular proliferation or increased apoptosis in this region (Figures 4A–D, I–K). At this stage of development, the size of the Col2a1-positive area, representing the Meckel’s cartilage, is similar control and *Neo1* ^*−/−*^ embryos (Figure 4K). While cellular proliferation was unchanged in the developing Meckel’s cartilage of *Neo1* ^*−/−*^ embryos, a subset of embryos analyzed (2/4) revealed an increased level of cellular apoptosis, which may contribute to the reduced Meckel’s cartilage size observed in E14.5 embryos (Figures 4E,F,J). Taken together, these data indicate that the transient increase in apoptosis in the mdBA1 mesenchyme and reduced osteoblastic differentiation of CNCCs observed in the mdBA1 of *Neo1* ^*−/−*^ embryos likely contribute to the mandibular bone development defects observed in these embryos.

### Neogenin ablation does not alter formation of the mesodermal core and mesoderm-derived blood vessels in the mdBA1

**FIGURE 5:**
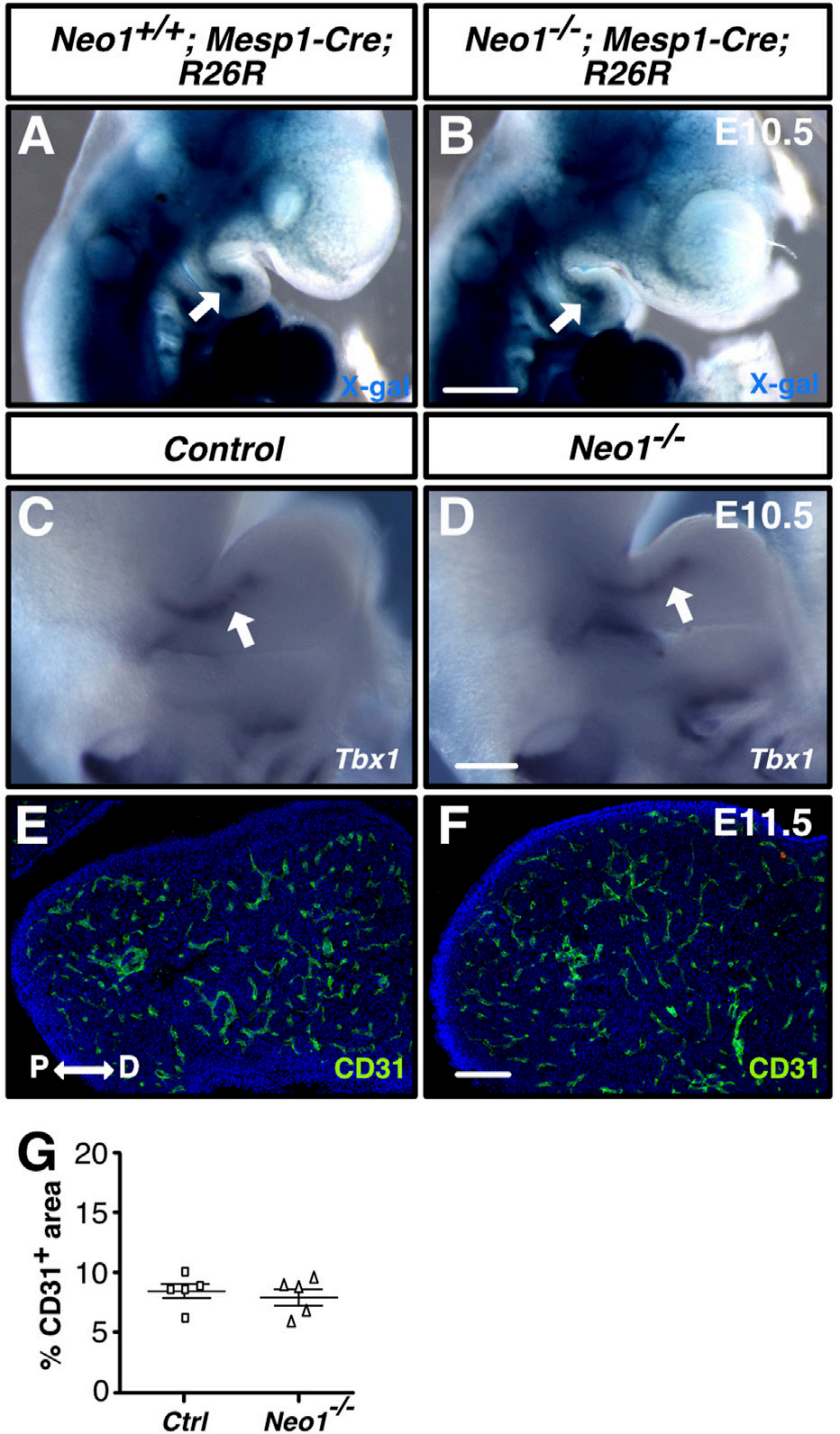
Development of the mdBA1 mesodermal core and blood vessels is unaffected in *Neo1* ^*−/−*^ embryos. **(A, B)** Whole-mount X-gal staining of E10.5 *Neo1* ^*+/+*^ *; Mesp1-Cre; R26R* **(A)** or *Neo1* ^*−/−*^ *; Mesp1-Cre; R26R* **(B)** embryos expressing β-galactosidase in mesoderm-derived cells. The mesodermal core (white arrow) is present in both *Neo1* ^*+/+*^ *; Mesp1-Cre; R26R* **(A)** and *Neo1* ^*−/−*^ *; Mesp1-Cre; R26R* **(B)** embryos (n = 4 *Neo1* ^*+/+*^ *; Mesp1-Cre; R26R-lacZ* and 4 *Neo1* ^*−/−*^ *; Mesp1-Cre; R26R-lacZ*). **(C, D)** Whole-mount *in situ* hybridization on E10.5 embryos using a *Tbx1* cRNA probe demonstrates that *Tbx1* is similarly expressed in the mesodermal core (white arrow) of the mdBA1 between control **(C)** and *Neo1* ^*−/−*^ **(D)** embryos (n = 6 controls and 3 *Neo1* ^*−/−*^). **(E, F)** Immunolabeling of transverse sections of the mdBA1 from E11.5 *Neo1* ^*+/+*^ **(E)** or *Neo1* ^*−/−*^ **(F)** with a CD31 antibody (green) and counterstaining with Hoechst 33,342 (blue). **(G)** Quantification of the percentage of mdBA1 area covered by CD31-positive blood vessels did not reveal any differences between control and *Neo1* ^*−/−*^ embryos (n = 5 controls and 5 *Neo1* ^*−/−*^, unpaired Student’s t-test, bars on graphs indicate mean ± s. e.m.). P: proximal; D: distal. Scale bars: 500 μm **(A, B)**, 250 μm **(C, D)**, 100 μm **(E, F)**.

The expression of Neogenin in the CM suggests that it may play a role in the formation of the mdBA1 core comprised of CM cells, commonly referred to as the mesodermal core, or for the development of other mesoderm-derived tissue, such as blood vessels, which have been implicated in regulating CNCC development (Wiszniak et al., 2015). We first determined whether formation of the mdBA1 mesodermal core is affected in *Neo1* ^*−/−*^ embryos by visualizing this structure using X-gal whole-mount labelling of E10.5 *Neo1* ^*−/−*^ *; Mesp1-Cre; R26R* embryos. The mesodermal core composed of β-galactosidase-positive cells appeared unchanged in *Neo1* ^*−/−*^ embryos (Figures 5A,B). We then examined whether expression of the transcription factor TBX1, whose expression in the mesodermal core is necessary for mandible development, may be altered in *Neo1* ^*−/−*^ embryos (Zhang et al., 2006; Aggarwal et al., 2010). The levels of *Tbx1* observed in control and *Neo1* ^*−/−*^ embryos appeared similar (Figures 5C,D). To assess whether formation of mesoderm-derived blood vessels in the mdBA1 is affected by loss of Neogenin, we measured the area of CD31-positive blood vessels in sections of mdBA1 from control and *Neo1* ^*−/−*^ embryos. A similar density of blood vessels was observed in the mdBA1 in these two populations of embryos (Figures 5E–G). Taken together, these results indicate that the reduced size of the mandible in *Neo1* ^*−/−*^ embryos is unlikely the result of improper development of the mesodermal core or reduced vascularization of the mdBA1.

### Spatial expression of *Bmp4* is altered in the mdBA1 ectoderm in *Neo1* ^*−/−*^ embryos

**FIGURE 6:**
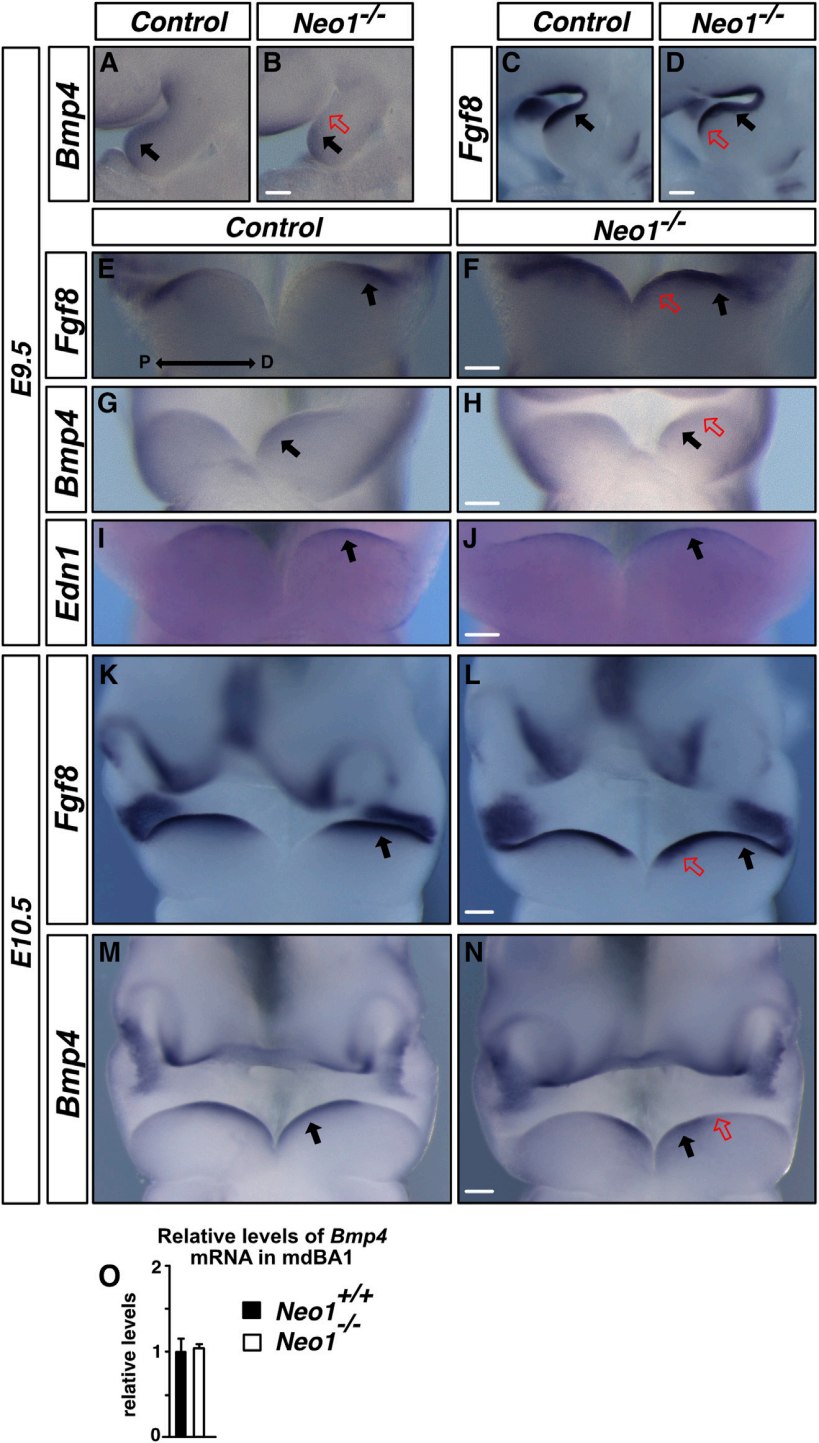
Loss of Neogenin leads to changes in expression of *Fgf8* and *Bmp4* in the mdBA1 ectoderm. **(A–N)** Examination of the expression of *Fgf8, Bmp4,* and *Edn1* in mdBA1 of E9.5 and E10.5 control and *Neo1^−/−^* embryos by *in situ* hybridization using cRNA probes. **(A–H)** *Fgf8* is expressed similarly in the proximal region (black arrows), but extends more distally in the mdBA1 (red open arrows in D and F) of *Neo1* ^*−/−*^ embryos. *Bmp4* is restricted to the distal region (black arrows), and is not observed in the proximal region in E9.5 *Neo1^−/−^* embryos (red open arrow in B and H). **(K–N)** These spatial differences in *Fgf8* and *Bmp4* expression persist at E10.5. **(I, J)** In contrast, similar patterns of expression of *Edn1* are observed in the mdBA1 ectoderm of control and *Neo1^−/−^* embryos. n > 3 for controls and ≥3 *Neo1^−/−^* embryos for each probe. P: proximal; D: distal. Scale bars: 125 μm **(A–D)**, 250 μm **(E–J)**, 125 μm **(K–N)**. **(O)** qRT-PCR quantification of relative levels of *Bmp4* mRNA at E10.5 in control and *Neo1^−/−^* mdBA1. (n = 3 control and 3 *Neo1^−/−^*, Student’s unpaired *t*-test, Bars on graphs indicate mean ± s. e.m.).

Several signalling molecules have been implicated in the regulation of cell survival and cell patterning within the developing mdBA1 mesenchyme, including FGF8, EDN1, and BMP4. Secretion of these molecules in the mdBA1 can affect the survival of CNCCs, as well as modulate the gene regulatory network that controls mdBA1 patterning. Since EDN1-mediated signalling can promote survival of mesenchymal cells (Clouthier et al., 2000; Abe et al., 2007), we examined *Edn1* expression by whole-mount *in situ* hybridization in *Neo1* ^*−/−*^ embryos at E9.5, a critical time for EDN1 signalling in the mdBA1 (Fukuhara et al., 2004; Ruest and Clouthier, 2009). Similar patterns of expression of *Edn1* were observed in the mdBA1 of control and *Neo1* ^*−/−*^ embryos at that stage of development (Figures 6I,J). FGF8, which is expressed by the ectoderm in the proximal region of the mdBA1, is necessary for the survival of CNCCs in this region of the mdBA1 (Trumpp et al., 1999). *Fgf8* expression was not reduced in the proximal region of the mdBA1 in *Neo1* ^*−/−*^ embryos at E9.5 and at E10.5, suggesting that a reduction in FGF8 signalling is unlikely to underlie the apoptosis observed in these embryos (Figures 6C–F, K,L). Interestingly, its expression extended more distally in the mdBA1 of *Neo1* ^*−/−*^ embryos indicating that regulatory mechanisms that control the spatial expression of *Fgf8* in the mdBA1 are affected in these embryos (Figures 6D,F,L). In contrast, the pattern of expression of *Fgf8* was unchanged in other embryo regions, such as the apical ectodermal ridge of the limb and the midbrain-hindbrain junction (data not shown).

A key modulator of FGF8 expression in the ectoderm is BMP4, whose presence in the mdBA1 ectoderm is necessary to restrict expression of FGF8 to the proximal region of the mdBA1 (Liu et al., 2005). Furthermore, regulation of either the levels of BMP4 or BMP signalling in the mdBA1 is especially critical for its proper development and for mandible outgrowth, as both excessive and reduced levels of BMP signalling antagonize mdBA1 development (Solloway and Robertson, 1999; Liu et al., 2005; Ko et al., 2007; Bonilla-Claudio et al., 2012). More specifically, ablation of BMP4 expression in the mdBA1 ectoderm or of the critical BMP signalling pathway component SMAD4 in neural crest cells both lead to increased apoptosis in the mdBA1 (Liu et al., 2005; Ko et al., 2007). An examination of the expression of *Bmp4* in the mdBA1 revealed that while the overall levels of *Bmp4* are unchanged, the ectodermal *Bmp4* expression is shifted towards the distal cap of the mdBA1 in the *Neo1* ^*−/−*^ embryos as early as E9.5 (Figures 6A,B,G,H,M,N,O). In contrast, the pattern of expression of *Bmp4* in some other regions of the embryo, such as the hindlimbs, is unchanged (data not shown). The altered expression of BMP4 along the proximo-distal axis may be responsible for the expansion in *Fgf8* expression to the distal part of the mdBA1 of *Neo1* ^*−/−*^ embryos. Furthermore, this observation suggests that altered expression of *Bmp4* when Neogenin is removed may affect expression of BMP signalling target genes that modulate the survival and development of cells within the mdBA1 mesenchyme.

### Reduced BMP signalling and expression of BMP target genes in the mdBA1 in the absence of Neogenin

**FIGURE 7:**
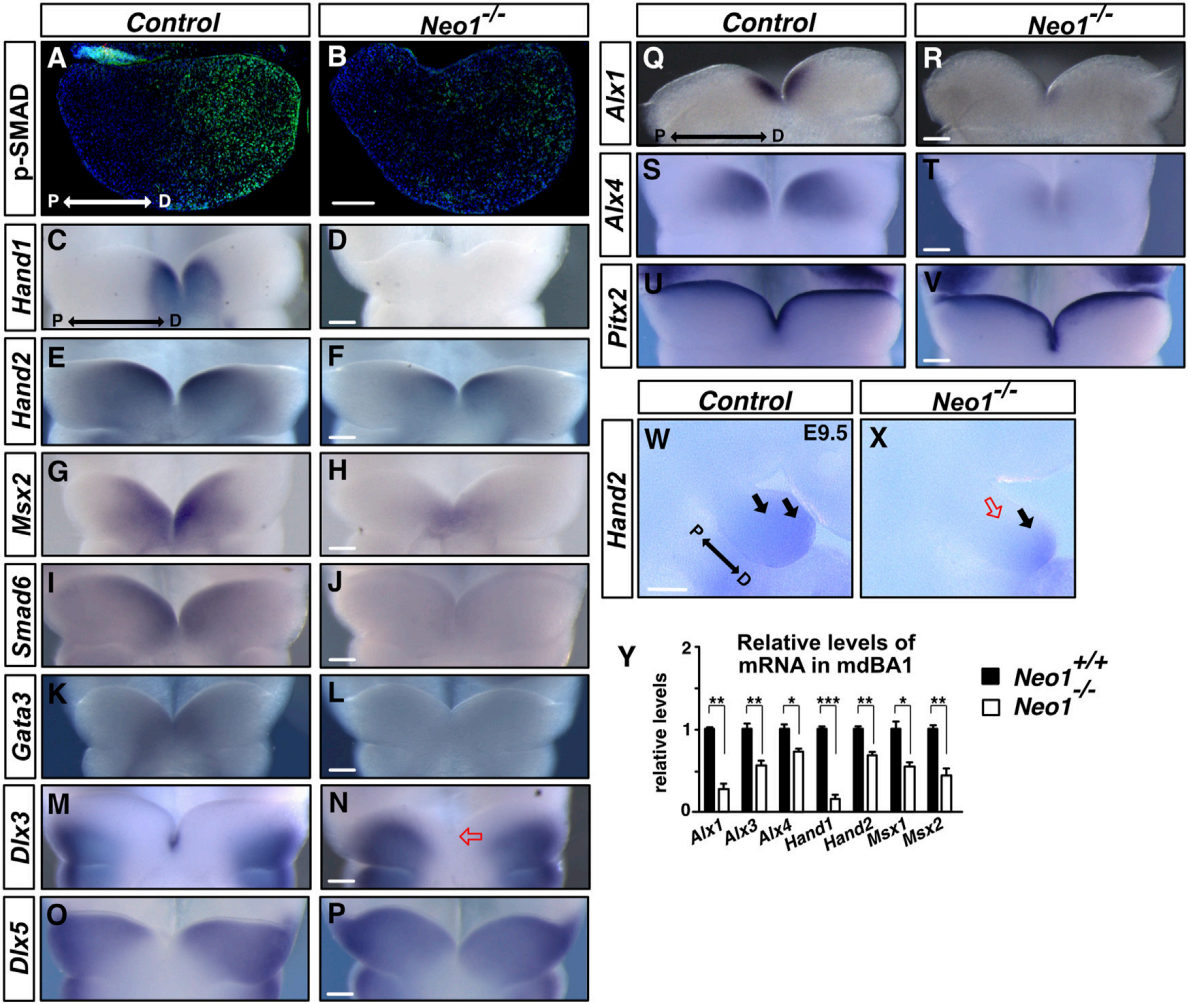
BMP signalling and expression of BMP target genes are decreased in the mdBA1 of *Neo1^−/−^* embryos. **(A, B)** Immunolabeling of transverse mdBA1 sections from control and *Neo1* ^−/−^ E10.5 embryos with Phospho-SMAD 1/5/8 antibodies (pSMAD, green) counterstained with Hoechst 33,342 (blue). In control embryos, a low proximal to high distal gradient of BMP signalling is observed, as previously described (Liu et al., 2005) **(A)**. Reduced pSMAD staining is observed in the mdBA1 of *Neo1^−/−^* embryos **(B)**. (n = 4 controls and 4 *Neo1^−/−^* embryos). **(C–T)** Examination of BMP target genes by whole mount *in situ* hybridization on the mdBA1 of E10.5 control and *Neo1^−/−^* embryos using *Hand1*, *Hand2*, *Msx2*, *Smad6*, *Gata3, Dlx3, Dlx5, Alx1*, and *Alx4* cRNA probes. *Hand1* expression is drastically reduced in *Neo1* ^−/−^ embryos **(C, D)**. Reduced expression of *Hand2* **(E, F)**, *Msx2* **(G, H)**, *Smad6* **(I, J)**, *Gata3* **(K, L)** *, Alx1* **(Q, R)**, and *Alx4* **(S, T)** is also observed in the mdBA1 of *Neo1* ^−/−^ embryos. The expression of *Dlx3* and *Dlx5* extends less distally in *Neo1* ^−/−^ embryos **(N, P)** and *Dlx3* is no longer expressed in the tip of the mdBA1 (red open arrow) (P) (n > 3 controls and ≥3 *Neo1* ^−/−^ embryos for each probe). **(U, V)** The pattern of expression of a non-BMP signalling target, *Pitx2*, is unchanged the mdBA1 of *Neo1* ^−/−^ embryos. **(W, X)** *Hand2* expression is also reduced in the proximal region of the mdBA1 at E9.5 in *Neo1* ^−/−^ embryos (red open arrow). **(Y)** qRT-PCR quantification of relative levels of *Alx1*, *Alx3*, *Alx4*, *Hand1*, *Hand2*, *Msx1*, and *Msx2* mRNA showed a reduction in the expression of these genes in the mdBA1 of E11.5 *Neo1* ^−/−^ embryos. (n = 4 control and *Neo1* ^*−/−*^ embryos, Student’s unpaired *t*-test, ***p < 0.001; **p < 0.01; *p < 0.05, Bars on graphs indicate mean ± s. e.m.). P: proximal; D: distal. Scale bars: 100 μm.

A proximo-distal gradient of BMP signalling can be observed in the mdBA1 by immunohistochemical detection of SMAD1/5/8 phosphorylation (pSMAD1/5/8), an essential step of BMP signalling, with the lowest levels of pSMAD1/5/8 observed in the proximal region and increasing towards the distal region (Figure 7A; Liu et al., 2005). Disruption of this BMP signalling gradient is associated with increased cellular apoptosis and improper patterning of the mdBA1 (Liu et al., 2005; MacKenzie et al., 2009). To examine whether the altered expression pattern of *Bmp4* in *Neo1* ^*−/−*^ embryos is associated with changes in BMP signalling in the mdBA1, we performed pSMAD1/5/8 immunostaining on sections of mdBA1 from E10.5 control and *Neo1* ^*−/−*^ embryos. We observed an overall reduction in the levels of BMP signalling in the mdBA1 in *Neo1* ^*−/−*^ embryos, with residual pSMAD staining being observed in the most distal region (Figures 7A,B).

To determine how decreased BMP signalling in the mesenchyme may affect development of the mdBA1, we examined the expression pattern of key BMP signalling-regulated genes that have been implicated in this process. We first assessed the expression of BMP target genes, such as members of the HAND, MSX, GATA, and SMAD family of transcription factors, some of which have been shown to be downregulated in the mdBA1 of mice lacking ectodermal BMP4 expression (Liu et al., 2005). HAND and MSX family transcription factors are necessary for mandible development, and their expression is BMP-dependent in the mdBA1 (Vainio et al., 1993; Satokata and Maas, 1994; Tucker et al., 1998; Yanagisawa et al., 2003; Barbosa et al., 2007; Firulli et al., 2014; Funato et al., 2015). Similarly, the transcription factor GATA3 is expressed in the most distal region of the mdBA1, it is necessary for jaw development, and its expression in the mdBA1 is BMP-dependent (Pandolfi et al., 1995; Ruest et al., 2004; Bonilla-Claudio et al., 2012). SMAD6 is also a direct target of BMP signalling which acts to antagonize BMP signalling, providing negative feedback of this pathway (Hata et al., 1998). At E10.5, *Hand1* expression in the distal part of the mdBA1 was drastically reduced in *Neo1* ^*−/−*^ embryos, while expression of *Hand2* was diminished, most notably in the proximal region (Figures 7C–F). The reduced expression of *Hand2* in the proximal region was also observed at E9.5, prior to the cellular apoptosis observed in this region at E10.5, indicating that reduced *Hand2* expression in this region is not due to loss of *Hand2*-expressing cells in *Neo1* ^*−/−*^ embryos (Figures 7W,X). *Msx2* expression was also decreased with residual expression in the most distal part of the arch in *Neo1* ^*−/−*^ embryos (Figures 7G,H). Furthermore, lower levels of *Smad6* and *Gata3* expression were detected in the distal mdBA1 of *Neo1* ^*−/−*^ embryos (Figures 7I–L). The decreased expression of *Hand* and *Msx* family genes in the mdBA1 of *Neo1* ^*−/−*^ embryos was also confirmed quantitatively by qRT-PCR analyses (Figure 7Y).

Regional specification along the proximo-distal axis of the mdBA1 relies in part on a combinatorial code of expression of *Dlx* family genes along this axis in the mdBA1 mesenchyme (Depew et al., 2002; Depew et al., 2005). Disruption of *Dlx* family genes leads to mandibular defects, and BMP signalling can modulate their patterns of expression. For example, BMP signalling is necessary to restrict Dlx5 expression to the proximal region of the mdBA1 (Vincentz et al., 2016). To examine whether loss of Neogenin alters expression of *Dlx* family members in the mdBA1, we examined the expression of *Dlx3* and *Dlx5* in control and *Neo1* ^*−/−*^ embryos. While *Dlx3* expression is restricted to the most proximal region and to the ectodermal cells in the tip of the mdBA1, *Dlx5* expression extends more distally without reaching the most distal part of the mdBA1 in control embryos (Figures 7M,O) (Depew et al., 2002). In contrast, *Dlx3* expression extends more distally and is lost in the mdBA1 distal tip of *Neo1* ^*−/−*^ embryos (Figure 7N). Consistent with the reduced BMP signalling observed in the mdBA1 of *Neo1* ^*−/−*^ embryos, *Dlx5* expression extends more distally in the mdBA1 of *Neo1* ^*−/−*^ embryos (Figure 7P).

BMP signalling within the mdBA1 also contributes to regulating the expression of the ALX family of transcription factors that control cell survival and jaw formation (Beverdam et al., 2001). Since ablation of BMP signalling in the mdBA1 mesenchyme through the specific deletion of *Smad4* in neural crest cells leads to loss of expression of members of the ALX family of transcription factors, we examined their expression in *Neo1* ^*−/−*^ embryos (Ko et al., 2007). A significant decrease in expression of *Alx1* and *Alx4* was observed in the distal region of the developing mdBA1 at E10.5 in the *Neo1* ^*−/−*^ embryos (Figures 7Q–T). The decreased expression of *Alx* family genes in the mdBA1 of *Neo1* ^*−/−*^ embryos was also confirmed quantitatively by qRT-PCR (Figure 7Y). While the pattern of expression of key BMP signalling targets is affected in the mdBA1 of *Neo1* ^*−/−*^ embryos, the expression of another transcription factor implicated in mdBA1 development, *Pitx2,* is unchanged, indicating that loss of Neogenin does not lead to a global change in gene transcription in the mdBA1 (Figures 7U,V). Hence, expression of several BMP signalling-dependent key transcription factors that modulate patterning of the mdBA1 during jaw development is altered in *Neo1* ^*−/−*^ embryos, which may contribute to the reduced mandible size observed in these embryos.

### Loss of Neogenin in the mesoderm results in craniofacial bone defects

**FIGURE 8:**
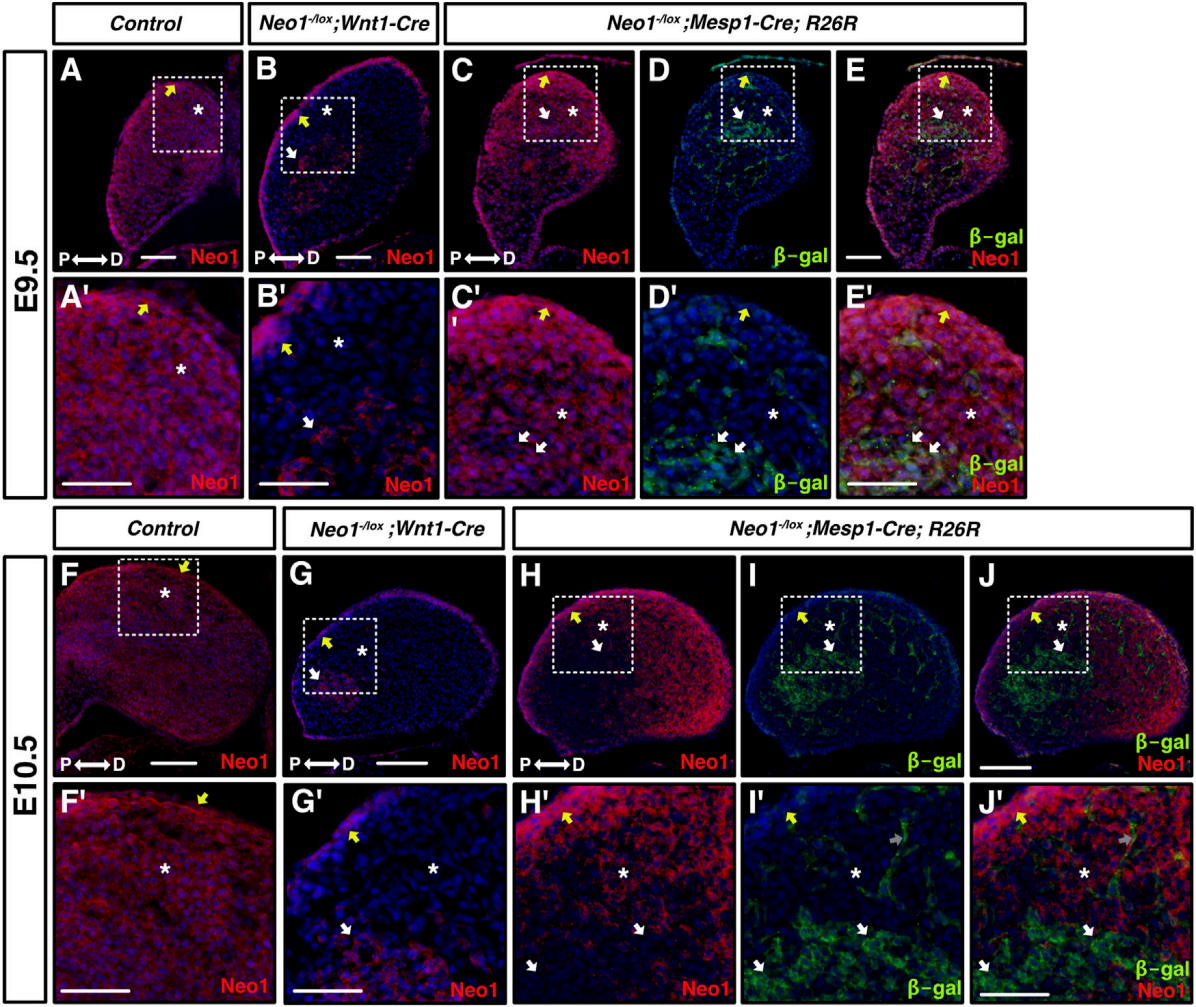
Cell-type specific ablation of Neogenin expression. **(A-J′)** Transverse sections of mdBA1 from E9.5 (top half of figure) and E10.5 (bottom half of figure) embryos expressing Cre in CNCCs- (*Wnt1-Cre*, **B, B′, G, G′**) and in mesoderm-derived cells (*Mesp1-Cre; R26R*, **C-E′** and **H-J′**), immunolabelled with antibodies against Neogenin (red) and β-galactosidase (green) and counterstained with Hoechst 33,342 (blue). Zoom-in views of mdBA1 show high levels of Neogenin expression in ectoderm (yellow arrow), CNCCs (white asterix), and mesoderm (white arrows) at both E9.5 and E10.5 **(A′-J′)**. In *Neo1* ^*-/lox*^ *; Wnt1-Cre* embryos, Neogenin signal is gone in CNCCs (white asterix) but remains present in ectoderm (yellow arrow) and mesoderm-derived cells (white arrow) at both embryonic stages examined **(B, B′, G, G′)**. Neogenin expression is specifically reduced at E9.5 and completely ablated at E10.5 from mesoderm-derived cells (white arrows) in *Neo1* ^*-/lox*^ *; Mesp1-Cre; R26R* embryos **(C-E′ and H-J′)**. P: proximal; D: distal. Scale bars: 50 μm **(A–E)**; 100 μm **(A′–E′)**; **(F–J)**; 200 μm **(F′–J′)**.

The dysregulation of gene expression in the mdBA1 of *Neo1* ^*−/−*^ embryos suggests that early defects in cell signalling in the developing mdBA1 may contribute to the reduced mandible size observed in older embryos. Since Neogenin is expressed in CNCCs and CM within the mdBA1, Neogenin may directly regulate the development of CNCCs or may be necessary in CM to modulate the expression of cues that influence CNCC development in the mdBA1. To examine how loss of Neogenin expression in these different cell types contributes to the craniofacial bone defects observed in *Neo1* ^*−/−*^ embryos, we ablated Neogenin expression using cell type-specific Cre recombination. Mice carrying a *Neo1* floxed allele were crossed with *Wnt1-Cre* and *Mesp1-Cre* mice expressing the Cre recombinase in neural crest and mesodermal cells, respectively, to ablate Neogenin expression in the CNCCs and mesoderm. At E9.5, Neogenin expression was specifically and completely ablated in the CNCCs of mdBA1 in *Neo1* ^*-/lox*^ *; Wnt1-Cre* embryos (Figures 8A–B’). In contrast, low levels of Neogenin expression could still be observed in subsets of CM cells at E9.5 in *Neo1* ^*-/lox*^ *; Mesp1-Cre* embryos, but its expression in these cells was completely lost by E10.5 (Figures 8C–E′, H-J′).

**FIGURE 9:**
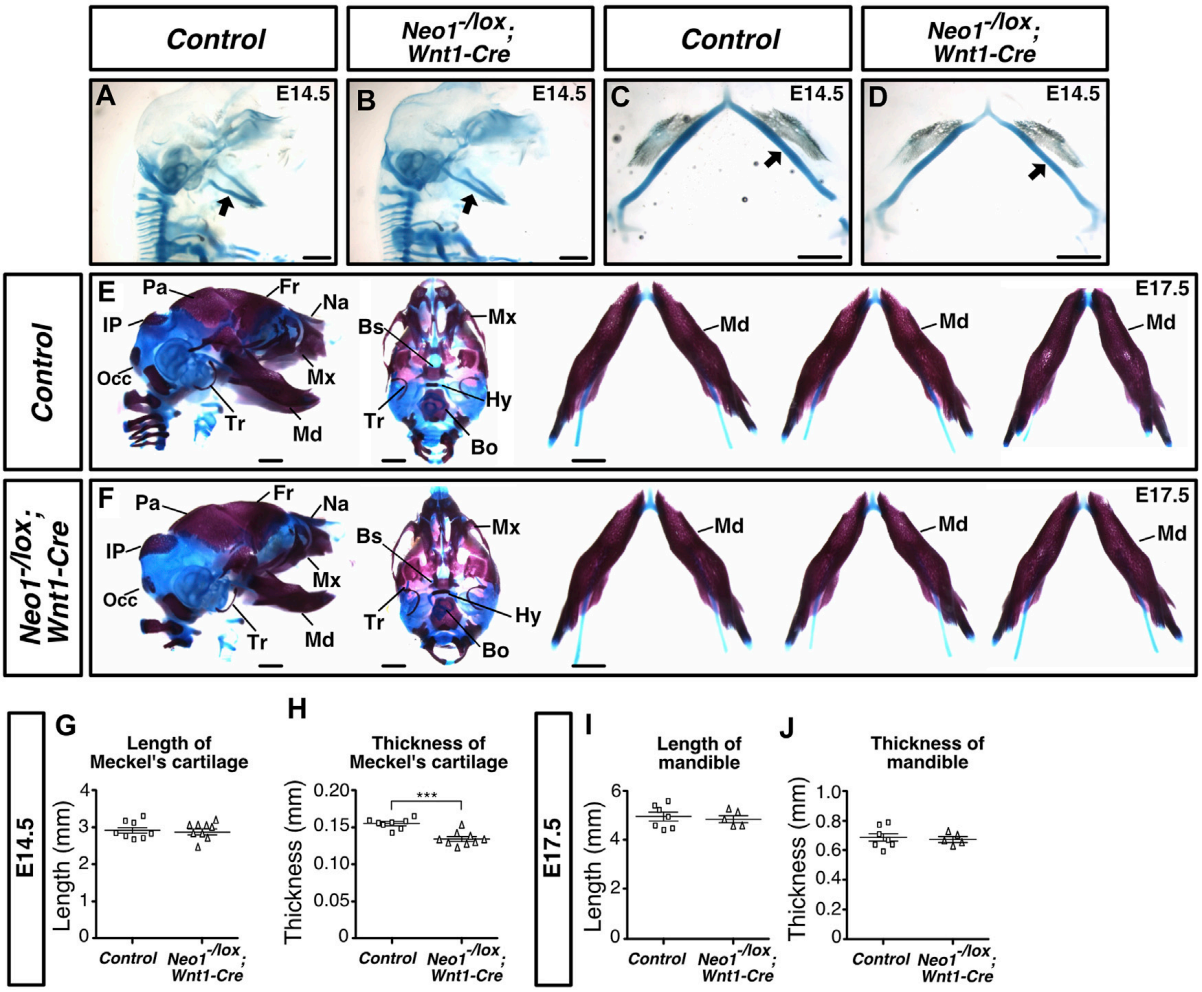
Ablation of Neogenin in CNCCs results in a transient craniofacial cartilage defect. **(A-D)** Alcian Blue (cartilage) staining highlights Meckel’s cartilage (arrow) in control **(A, D)** and *Neo1* ^*-/lox*^ *; Wnt1-Cre* **(B, C, E)** embryos at E14.5, and when quantified revealed a decrease in the thickness, but not the length of this structure in conditional knock-out embryos [**(H, G)**, respectively]. (n = 8 controls; n = 9 *Neo1* ^*-/lox*^ *; Wnt1-Cre;* Student’s unpaired *t*-test, ***p < 0.001, Bars on graphs indicate mean ± s. e.m.). **(E, F)** Alizarin Red (bone) and Alcian Blue (cartilage) staining of E17.5 control **(E)** and *Neo1^-/lox^*; *Wnt1-Cre* **(F)** embryos with lateral (left) and ventral (middle) views of the skull, and dissected mandibles (right). No mandible size defects were observed in *Neo1* ^*-/lox*^ *; Wnt1-Cre* embryos, as shown in three representative embryos (right) and quantified in **(I)** and **(J)** (n = 7 controls; n = 5 *Neo1* ^*-/lox*^ *; Wnt1-Cre* quantified, Bars on graphs indicate mean ± s. e.m.). Bones, or their expected location when missing, are labelled as follows: Bo: basioccipital, Bs: basisphenoid, Fr: frontal, Hy: hyoid, Ip: interparietal, Md: mandible, Mx: maxillary, Na: nasal bone, Occ: occipital, Pa: parietal, Tr: tympanic ring; P: proximal, D: distal. Scale bars: 1 mm.

**FIGURE 10:**
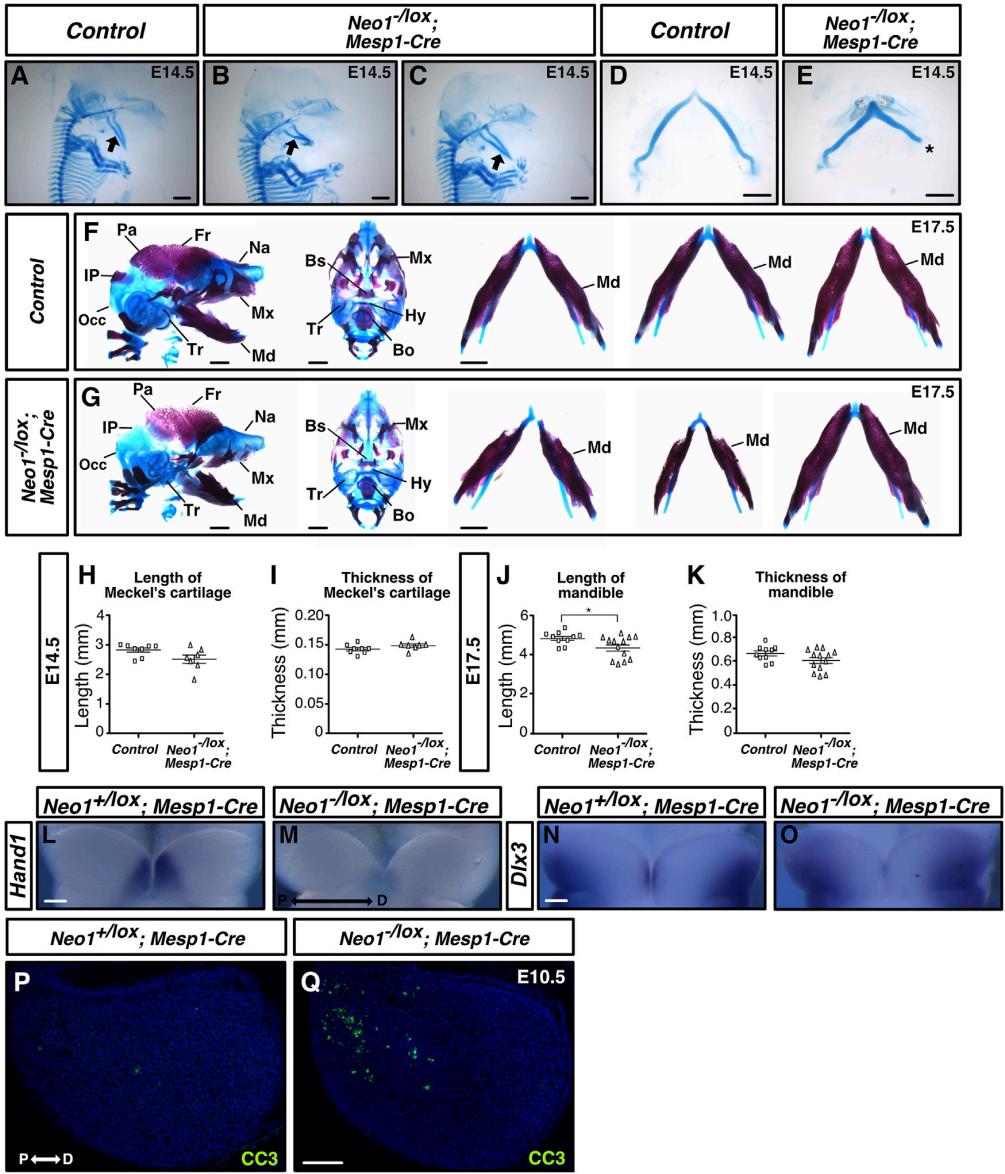
Ablation of Neogenin in mesodermal cells leads to craniofacial bone defects. **(A–E)** Alcian Blue (cartilage) staining highlights Meckel’s cartilage in control and *Neo1* ^*-/lox*^ *; Mesp1-Cre* embryos at E14.5, and when quantified revealed a subset of conditional knockout embryos with a shorter Meckel’s cartilage (1/7 embryos examined), without a change in thickness [**(H, I)**, respectively]. Asterisk in E indicates damage from dissection of Meckel’s cartilage. (n = 8 controls; n = 7 *Neo1* ^*-/lox*^ *; Mesp1-Cre;* Bars on graphs indicate mean ± s. e.m.). **(F, G)** Alizarin Red (bone) and Alcian Blue (cartilage) staining of E17.5 control (F) and *Neo1* ^*-/lox*^ *; Mesp1-Cre* conditional knock-out (G) embryos with lateral (left) and ventral (middle) views, and dissected mandible (right). (G) In *Neo1* ^*-/lox*^ *; Mesp1-Cre* embryos, the lateral view (left) shows a reduction in mandible length and thickness (4/13 embryos examined) and an improper formation of additional CNCC-derived bones, including the nasal, maxillary, and tympanic bones. The view of the dissected mandible from three representative embryos (right) shows the phenotypic variability observed in mandible size. Reduced size of the mesoderm-derived parietal, interparietal, and occipital bones is also observed in these embryos. **(J, K)** Quantification of mandible length and thickness at E17.5 reflects the size defects observed in a subgroup of these conditional knockouts. (n = 10 controls and 13 *Neo1* ^*-/lox*^ *; Mesp1-Cre;* Student’s unpaired *t*-test, *p < 0.05, Bars on graphs indicate mean ± s. e.m.). Bones, or their expected location when missing, are labelled as follows: Bo: basioccipital, Bs: basisphenoid, Fr: frontal, Hy: hyoid, Ip: interparietal, Md: mandible, Mx: maxillary, Na: nasal bone, Occ: occipital, Pa: parietal, Tr: tympanic ring. **(L–O)** Whole-mount *in situ* hybridization on the mdBA1 of E10.5 control and *Neo1^-/lox^; Mesp1-Cre* conditional knock-out embryos using *Hand1* (L, M) and *Dlx3* (N, O) cRNA probes. The expression of *Hand1* and *Dlx3* is altered in *Neo1^-/lox^; Mesp1-Cre* (1/3 embryos examined for each cRNA probe) **(P, Q)** Immunolabeling for the cell death marker cleaved-caspase 3 (CC3) on mdBA1 sections from E10.5 control and *Neo1^-/lox^; Mesp1-Cre* embryos. In a subset of conditional knock-out embryos (3/6 embryos examined), we observed an increase in CC3 labeled cells in the proximal region of the mdBA1 **(Q)** P: proximal, D: distal. Scale bars: 1 mm **(A–G)**, 100 μm **(L–Q)**.

Cartilage staining at E14.5 revealed that the Meckel’s cartilage was thinner in *Neo1* ^*-/lox*^ *; Wnt1-Cre* embryos, while its length was unchanged (Figures 9A–D, G,H). However, by E17.5, skeletal staining using Alcian blue and Alizarin red did not reveal discernible craniofacial bone defects in *Neo1* ^*-/lox*^ *; Wnt1-Cre* embryos (Figures 9F,I,J). In contrast, subsets of *Neo1* ^*-/lox*^ *; Mesp1-Cre* embryos showed craniofacial cartilage and bone defects with variable penetrance at E14.5 and E17.5, respectively (Figure 10). The length, but not the thickness, of Meckel’s cartilage was reduced in some *Neo1* ^*-/lox*^ *; Mesp1-Cre* embryos (2/7) at E14.5 (Figures 10B–E,H,I). At E17.5, Some *Neo1* ^*-/lox*^ *; Mesp1-Cre* embryos displayed reduced size of the parietal bone (6/13), reduced size or absence of the interparietal bone (7/13), a shorter mandible (4/13), and absence of a nasal bone (6/13), while the rest of the embryos analyzed (6/13) did not display any distinguishable craniofacial bone defects (Figures 10G,J,K).

To determine whether the pattern of expression of BMP target genes is affected in the mdBA1 of *Neo1* ^*-/lox*^ *; Mesp1-Cre*, we examined the expression of *Hand1* at E10.5, as it was the most drastically affected BMP target in the mdBA1 of *Neo1* ^*−/−*^ embryos. We observed that a subset of *Neo1* ^*-/lox*^ *; Mesp1-Cre* embryos (1/3) showed a reduction in the expression of *Hand1* within the distal region of the mdBA1 (Figures 10L,M). The pattern of expression of *Dlx3* was also affected in a subset of *Neo1* ^*-/lox*^ *; Mesp1-Cre* embryos (1/3) we analyzed (Figures 10N,O). Furthermore, as observed in *Neo1* ^*−/−*^ embryos, increased apoptosis in the proximal region of the mdBA1 was observed in some *Neo1* ^*-/lox*^ *; Mesp1-Cre* embryos (3/6) (Figures 10P,Q). Overall, these results indicate that loss of Neogenin expression in the mesoderm, at least in part, contributes to the craniofacial bone defects observed in *Neo1* ^*−/−*^ embryos.

## Discussion

### Neogenin is required for craniofacial development

Our studies identify an as-of-yet unrecognized function for the transmembrane receptor Neogenin in regulating mdBA1 development by influencing several processes, including cell survival, CNCC differentiation, and the expression of BMP-dependent tissue patterning genes. We found that ablation of Neogenin expression also leads to improper development of multiple bones of the skull that display phenotypic variability across *Neo1^−/−^* mice, as seen with the mandibular defects ranging from subtle micrognathia to complete agnathia. While decreased expression of BMP signalling-dependent genes, such as *Hand1*, was observed in all *Neo1^−/−^* embryos analyzed, the levels of apoptosis observed in the mdBA1 of *Neo1^−/−^* embryos at E10.5 were variable (Figure 3H), suggesting that differences in apoptosis levels could in part contribute to the variability observed in mandible size and in the development of other bones of the skulls in these *Neo1^−/−^* embryos. We have previously shown, in the developing olfactory epithelium, another instance of variability in which levels of cellular apoptosis were mirrored by variability in phenotypic defects observed in *Neo1^−/−^* embryos (Kam et al., 2016).

### Reduced BMP signalling and increased apoptosis in the mdBA1 of *Neo1^−/−^* embryos

Loss of Neogenin leads to increased cellular apoptosis and decreased osteoblastic differentiation in the mdBA1 of *Neo1^−/−^* embryos. This increased apoptosis may reduce the number of CNCCs available for differentiation into osteoblasts. Alternatively, CNCCs may fail to properly differentiate into osteoblasts, resulting in increased numbers of CNCCs undergoing cell death. Either of these two possible scenarios would ultimately lead to a decrease in mandibular bone volume, as observed in *Neo1^−/−^* embryos. The apoptosis, which was restricted to the proximal region of the mdBA1, appeared only at E10.5, and was transient as no significant change in apoptosis was observed at E11.5. It is possible that late arriving CNCCs, located more proximally in the mdBA1, are more sensitive to the changes in gene expression observed in the mdBA1 following Neogenin ablation. In chick, CNCCs in the distal and proximal regions of the mdBA1 have different molecular signatures, suggesting they may respond differently to environmental signals in this structure (Morrison et al., 2017). Although outgrowth of the mandible takes place at the distal end of the mdBA1, apoptosis in the proximal region of the mdBA1 may in part contribute to the reduced size of the mandible in *Neo1^−/−^* embryos. For example, a specific increase in apoptosis in the proximal region of the developing arch/mandible has been associated with reduced mandible size in mice lacking Alk5 expression in CNCCs, indicating that cell death in this region of the mdBA1 can have a detrimental effect on mandible outgrowth (Zhao et al., 2008).

The tight spatio-temporal regulation of BMP signalling in the developing mdBA1 is crucial to modulate the gene regulatory network necessary for mdBA1 patterning, mesenchyme cell survival and proliferation, and osteoblast differentiation. Reduced or increased BMP signalling have both been reported to lead to micrognathia in mouse embryos (Solloway and Robertson, 1999; Liu et al., 2005; Ko et al., 2007; Bonilla-Claudio et al., 2012). It is therefore likely that multiple mechanisms are in place to modulate BMP signalling in the mdBA1, including expression of molecules that enhance and inhibit BMP signalling. We found that Neogenin is required to maintain the proximo-distal gradient of BMP signalling in the mdBA1 and that its ablation leads to reduced expression of several BMP target genes in the mdBA1, including members of the HAND and MSX transcription factor families (Figure 7). Disruption of the proximo-distal gradient of BMP signalling is likely related to the shift observed in *Bmp4* expression in the ectoderm of *Neo1^−/−^* embryos. Indeed, in these embryos, *Bmp4* expression in the ectoderm is restricted to the distal region as early as E9.5. Alternatively, loss of Neogenin may lead to a spatial change or an enhancement in the expression of secreted molecules that inhibit BMP signalling, such as Noggin, Chordin, and TWSG1, which are expressed in the mdBA1 and could modulate the formation of the BMP signalling gradient in the mdBA1 (Stottmann et al., 2001; MacKenzie et al., 2009).

Reduced BMP signalling may also underlie the expansion in expression of *Dlx3* and *Dlx5* from the proximal to more distal part of the mdBA1 observed in *Neo1^−/−^* embryos, which suggests that the proximo-distal patterning of the mdBA1 is affected in the absence of Neogenin. Reduced BMP signalling in CNCCs by specific ablation of Smad4 leads to a similar expansion of Dlx5 expression within the mdBA1 (Ko et al., 2007). While we cannot exclude the possibility that the altered expression of *Fgf8* in the ectoderm of *Neo1^−/−^* embryos also contributes to proximo-distal patterning defects in these embryos, its previous ablation in the ectoderm did not lead to changes in the patterns of expression of *Dlx* genes in the mdBA1, making it unlikely that FGF8 plays a role in this specific process (Trumpp et al., 1999).

### Mesodermal expression of Neogenin regulates the development of both CM- and CNCC-derived craniofacial structures

As Neogenin is expressed in several populations of cells with different embryonic origins that together make up important precursors of craniofacial structures, it may contribute to craniofacial development in both cell- and non-cell autonomous ways. A cell-autonomous role for Neogenin in neural crest cells has previously been shown in the formation of the retrolental mass of the eye in mice (Lin et al., 2020). Our studies have revealed that loss of Neogenin expression in CNCCs leads to reduced thickness of the developing mandible cartilage at E14.5, but does not result in any mandibular bone defects by E17.5. In contrast, loss of Neogenin in the mesoderm results in defects in both CM- and CNCC-derived craniofacial bones indicating that it regulates more than one cellular process important in, or preceding, craniofacial bone development via different molecular mechanisms. Since some *Neo1* ^*-/lox*^ *; Mesp1-Cre* embryos display similar CM-derived bone formation defects to the *Neo1^−/−^* embryos, such as a poorly developed parietal bone, we postulate that Neogenin could function to cell-autonomously enhance BMP signalling in mesenchymal progenitor cells for the development of these bones. Neogenin has previously been reported to contribute to endochondral ossification of long bones by enhancing BMP-dependent chondrogenesis (Zhou et al., 2010). In this process, Neogenin enhances BMP signalling in chondrocytes by promoting the localization of BMP receptors to membrane microdomains. The recruitment of BMP receptors to these microdomains requires an association between Neogenin and the GPI-anchored protein RGMb, which acts as a BMP co-receptor and enhances BMP binding to their receptors (Zhou et al., 2010). Alternatively, Neogenin signalling may regulate CM-derived bone development by acting as a receptor for BMPs. Several BMPs have been reported to bind directly to Neogenin and its knockdown in the mesoderm-derived cell line C2C12 leads to increased SMAD phosphorylation, indicating that Neogenin signalling can inhibit BMP signalling in these cells (Hagihara et al., 2011). However, we did not observe an increase in pSMAD immunohistochemical signal in the mesodermal core of the mdBA1 of *Neo1^−/−^* embryos, suggesting that Neogenin does not inhibit BMP signalling in cranial mesoderm *in vivo*.

The cell type-specific Neogenin ablation analyses have also revealed that Neogenin regulates the formation of the CNCC-derived mandible, as well as nasal and maxillary bones, through a non-cell autonomous mechanism as the specific ablation of Neogenin in neural crest cells did not grossly affect the formation of these craniofacial bones, demonstrating that it is dispensable in these cells for craniofacial bone development. On the other hand, a subset of *Neo1^-/lox^; Mesp1-Cre* embryos displayed similar craniofacial phenotypes as *Neo1^−/−^* embryos, including micrognathia and improper development of CNCC-derived bones, indicating that Neogenin expression in the CM contributes to the formation of the mandible. Although we did not see any defects in the formation of mesoderm-derived structures themselves within the mdBA1 in embryos lacking Neogenin, such as the vasculature or the mesodermal core, we cannot exclude the potential effects of removing Neogenin expression from mesoderm-derived tissues outside the mdBA1 on the development of the mandible. Additionally, the cranial mesoderm gives rise to muscle tissue within the developing jaw. Cellular interactions between developing muscle tissue and developing skeletal structures are well-documented and have been shown to influence the formation of both bone and muscle (Buvinic et al., 2021). These interactions can include both molecular cell-signalling, as well as the mutual exertion of mechanical forces between these two tissues (Brotto and Bonewald, 2015; Sunadome et al., 2023). Interestingly, Neogenin has previously been implicated in the regulation of myofiber size through its interaction with two different ligands, netrin and RGMa, and may therefore modulate muscle formation in the craniofacial skeleton (Kang et al., 2004; Bae et al., 2009; do Carmo Costa et al., 2021).

The lower penetrance of the mandibular phenotype observed in *Neo1^-/lox^; Mesp1-Cre* embryos may be related to the timing of Neogenin ablation in the CM. While Neogenin protein is completely absent in the CM at E10.5, residual expression was observed at E9.5, a key time point in mdBA1 patterning. Hence, the levels of Neogenin in the CM at E9.5 may differ across *Neo1^-/lox^; Mesp1-Cre* embryos, leading to different phenotypic outcomes. It remains also possible that Neogenin expression in the ectoderm or endoderm contributes to mandible development and that its ablation in multiple populations of cells would be necessary to fully recapitulate the penetrance of the phenotypes observed in *Neo1^−/−^* embryos.

**FIGURE 11:**
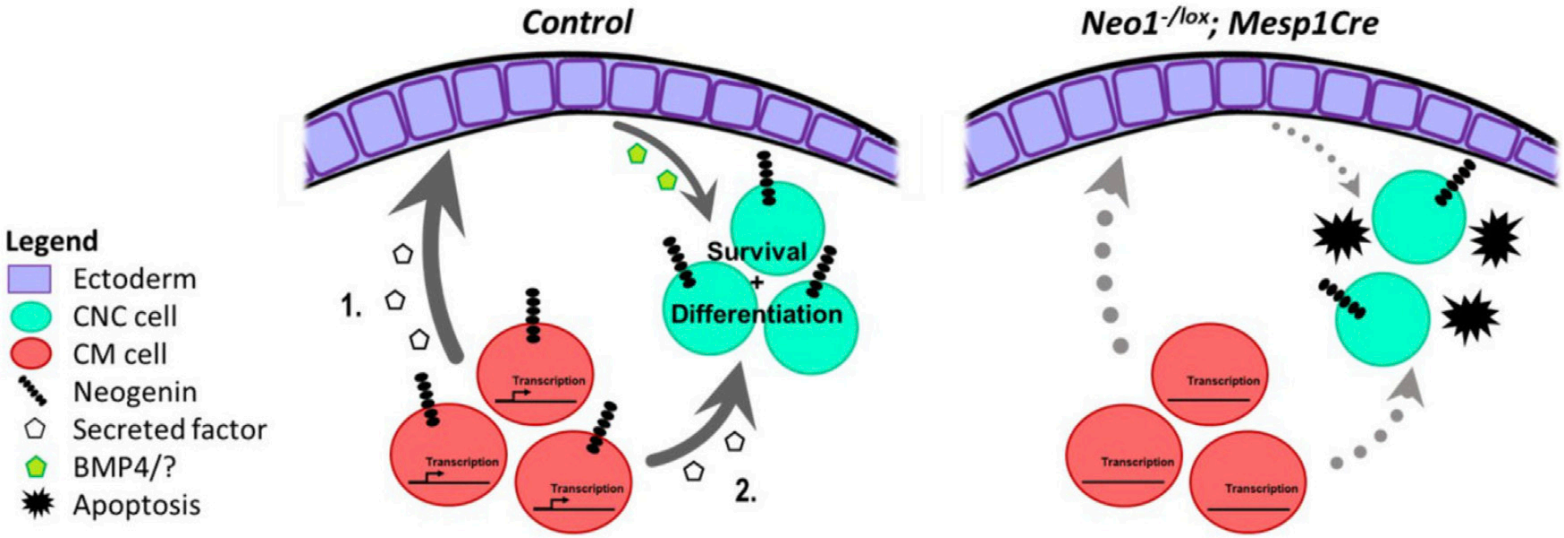
Potential molecular mechanisms for Neogenin function in the developing mdBA1. During development Neogenin expression in CM cells may regulate expression of a CM-derived secreted factor that affects mdBA1 development. This CM-derived molecule could promote ectodermal expression of proteins, such as BMP4, that influence the survival and possibly differentiation of CNC cells. (1.). Alternatively, the Neogenin-regulated CM-derived factor may directly impinge on the development of CNC cells. (2.). Loss of Neogenin in CM cells in *Neo1* ^*-/lox*^ *;Mesp1-Cre* embryos would alter expression of the CM-derived factor leading to increased apoptosis and reduced differentiation of CNC cells.

Based on our analyses, we propose that Neogenin signalling in the CM contributes to the expression of secreted molecules that directly or indirectly modulate CNCC survival and patterning during mdBA1 development, possibly by influencing mdBA1 epithelial cell expression of factors, such as BMP4 (Figure 11). In keeping with this model, our *in situ* hybridization analyses revealed a spatial shift in *Bmp4* expression in the mdBA1 ectoderm in *Neo1^−/−^* embryos as well as a decrease in BMP signalling in the mdBA1 (Figures 6, 7). Alternatively, Neogenin may regulate the expression of CM-derived factors that directly affect CNCC development through BMP signalling (Figure 11). For example, microarray-based gene expression analyses in E9.5 mouse embryos identified BMP4 as a candidate CM-derived signal in the development of CNCCs in the mdBA1 (Fan et al., 2016). In both cases, loss of Neogenin in CM cells would result in altered expression of CM-derived factors that ultimately leads to reduced BMP signalling in the mdBA1 mesenchyme, improper expression of patterning genes, increased apoptosis in the mdBA1, and improper osteoblastic differentiation.

Neogenin may regulate expression of CM-derived factors by directly modulating gene expression in these cells. Indeed, the intracellular domain of Neogenin can undergo cleavage to translocate to the nucleus and regulate gene expression (Goldschneider et al., 2008). Translocation of its intracellular domain to the nucleus is necessary for neural tube morphogenesis in *Xenopus*, indicating it can regulate physiological processes through modulation of gene expression (Brown et al., 2019). The future discovery of specific target genes for the intracellular domain of Neogenin in mesodermal cells could identify CM-derived molecules with previously unrecognized function in modulating CNCC development and will help further define Neogenin’s mode of action in craniofacial development.

## Data Availability

The raw data supporting the conclusions of this article will be made available by the authors, without undue reservation.
