# Supplementary material for: Loss of Neogenin alters branchial arch development and leads to craniofacial skeletal defects
Source: Front Cell Dev Biol. 2024 Feb 9;12:1256465. doi: 10.3389/fcell.2024.1256465 (PMC10884240; doi:10.3389/fcell.2024.1256465)
Supplement: Supplementary file 1 [file DataSheet1.pdf]

Quilez et al.

Supplemental Information

Supplemental Methods:

Analysis of dorsal root ganglia.

E15.5 embryos were dissected, immersion fixed for 45 minutes in 4% paraformaldehyde (PFA), and transverse 20 µm sections were processed for immunohistochemistry using conditions previously described (Cho et al., 2012). Sections were incubated at 4^0C overnight with a NeuN primary antibody (1:1000; Millipore MAB377). After rinsing in PBS, primary antibody binding was detected with the appropriate Alexa-488 secondary antibody (1:500) (Invitrogen). Sections were then washed in 1x PBS and incubated with Hoechst (Molecular Probes). Slides were mounted with Fluoromount-G (Southern Biotech). Images of processed sections were obtained using epifluorescence microscopy with a Carl Zeiss Axio Imager M1. Quantification of cell number and DRG area was performed using Icy software or ImageJ. Alternating sections were collected on ten individual slides starting at the forelimb and ending at the hindlimbs. One slide (10 sections) per embryos was quantified and the measurements were averaged for each embryo.

Supplemental Data:

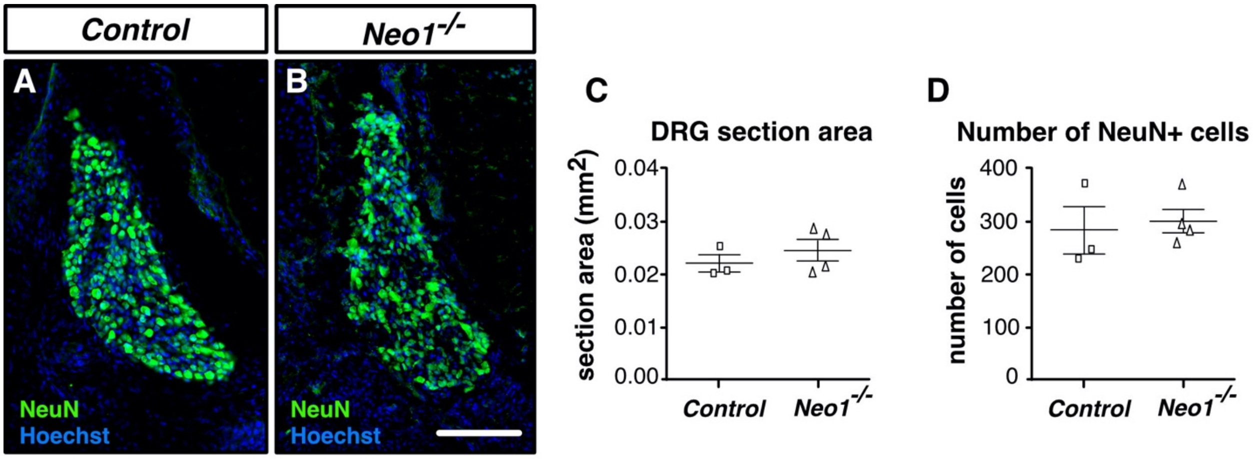

Figure S1: Formation of the dorsal root ganglia is unaffected in Neo1$^{-/-}$ embryos.

(A,B) Sections of dorsal root ganglia from control and *Neo1$^{-/-}$* E14.5 embryos were labelled with NeuN antibodies to identify neurons and with Hoechst to visualize cell nuclei. The average area of DRGs and the number of NeuN-positive cells were similar in control and *Neo1$^{-/-}$* embryos. (controls: n=3; Neo1-/-: n= 4) Student's unpaired t-test, Bars on graphs indicate mean± s.e.m. Scale bar: 100 µm

	Mandible defect	Hypomorphic liver	Oedema	Exencephaly	Cleft Palate
Neo1$^{+/+}$ **or** $^{+/-}$	0% (0/192)	0% (0/192)	0% (0/192)	0% (0/192)	0% (0/15)
Neo1$^{-/-}$	77% (54/70)	67% (47/70)	29% (20/70)	7% (5/70)	80% (4/5)

Table S1. Penetrance of phenotypes observed in *Neo1*$^{-/-}$ embryos.
Summary of the penetrance of four phenotypes observed in dissected E14.5 and older embryos.

	**E10.5**	**E11.5**	**E13.5**	**E14.5**	**E15.5**	**E16.5**
**Means CRL+/-SEM**	mm (n)	mm (n)	mm (n)	mm (n)	mm (n)	mm (n)
Neo1$^{+/+}$	4.88+/-0.08 (8)	6.39+/-0.17 (35)	9.25+/-0.11 (5)	12.86+/-0.29 (11)	14.40+/-0.11 (44)	15.93+/-0.19 (21)
Neo1$^{+/-}$	5.05+/- 0.05(11)	6.65+/-0.10 (93)	9.38 +/-0.15(10)	12.47+/-0.22 (18)	14.32+/-0.07 (92)	16.16+/- 0.12(44)
Neo1$^{-/-}$	5.00+/-0.00 (5)	6.68+/-0.14 (41)	9.29+/-0.11 (7)	11.47+/- 0.19(15)	13.27+/-0.12 (39)	14.75+/-0.24 (16)
**ANOVA P-Value**	0.1157	0.3074	0.8189	**0.0004**	**5.20E-13**	**8.91E-07**

Table S2. Size of control and *Neo1*$^{-/-}$ embryos at different stages of development.
Crown-rump lengths (CRL) of freshly dissected embryos at E10.5, E11.5, E13.5, E14.5, E15.5, and E16.5. The average length +/- standard error of the mean (SEM) and the number of measured embryos (n) are indicated in the table. Embryo lengths for each stage were compared using a One-way ANOVA test and the resulting P-values are indicated in the table. A significant decrease in the size of *Neo1*$^{-/-}$ embryos was observed starting at E14.5.
